# *In vitro* and *in vivo* activities of KSP-1007, a broad-spectrum inhibitor of serine- and metallo-β-lactamases, in combination with meropenem against carbapenem-resistant Gram-negative bacteria

**DOI:** 10.1128/aac.01602-23

**Published:** 2024-05-06

**Authors:** Koji Takemoto, Ryo Nakayama, Koichi Fujimoto, Yumiko Suzuki, Yukiko Takarabe, Masako Honsho, Sachiko Kitahara, Yoshihiko Noguchi, Hidehito Matsui, Tomoyasu Hirose, Yukihiro Asami, Jun Hidaka, Toshiaki Sunazuka, Hideaki Hanaki

**Affiliations:** 1 Drug Research Division, Sumitomo Pharma Co., Ltd., Osaka, Japan; 2 Ōmura Satoshi Memorial Institute, Kitasato University, Tokyo, Japan; 3 Graduate School of Infection Control Sciences, Kitasato University, Tokyo, Japan; University of Fribourg, Fribourg, Switzerland

**Keywords:** beta-lactamases, carbapenems, antibiotic resistance

## Abstract

KSP-1007 is a novel bicyclic boronate-based broad-spectrum β-lactamase inhibitor and is being developed in combination with meropenem (MEM) for the treatment of infections caused by carbapenem-resistant Gram-negative bacteria, a global health concern, and here, we describe its characteristics. KSP-1007 exhibited low apparent inhibition constant (*K_i_*_app_) values against all classes of β-lactamase, including imipenemase types and oxacillinase types from *Acinetobacter baumannii*. Against 207 *Enterobacterales* and 55 *A*. *baumannii*, including carbapenemase producers, KSP-1007 at fixed concentrations of 4, 8, and 16 µg/mL dose-dependently potentiated the *in vitro* activity of MEM in broth microdilution MIC testing. The MIC_90_ of MEM/KSP-1007 at 8 µg/mL against *Enterobacterales* was lower than those of MEM/vaborbactam, ceftazidime/avibactam, imipenem/relebactam, and colistin and similar to those of aztreonam/avibactam, cefiderocol, and tigecycline. The *in vitro* activity of MEM/KSP-1007 at ≥4 µg/mL against *Enterobacterales* harboring metallo-β-lactamase was superior to that of cefepime/taniborbactam. MEM/KSP-1007 showed excellent activity against *Escherichia coli* with PBP3 mutations and New Delhi metallo-β-lactamase compared to aztreonam/avibactam, cefepime/taniborbactam, and cefiderocol. MEM/KSP-1007 at 8 µg/mL showed greater efficacy against *A. baumannii* than these comparators except for cefiderocol, tigecycline, and colistin. A 2-fold reduction in MEM MIC against 96 *Pseudomonas aeruginosa* was observed in combination with KSP-1007. MEM/KSP-1007 demonstrated bactericidal activity against carbapenemase-producing *Enterobacterales*, *A. baumannii*, and *P. aeruginosa* based on minimum bactericidal concentration/MIC ratios of ≤4. KSP-1007 enhanced the *in vivo* activity of MEM against carbapenemase-producing *Enterobacterales*, *A. baumannii*, and *P. aeruginosa* in murine systemic, complicated urinary tract, and thigh infection models. Collectively, MEM/KSP-1007 has a good profile for treating carbapenem-resistant Gram-negative bacterial infections.

## INTRODUCTION

The emergence of carbapenem-resistant Gram-negative bacteria (CR-GNB), such as carbapenem-resistant *Enterobacterales* (CRE), *Acinetobacter baumannii* (CRAB), and *Pseudomonas aeruginosa* (CRPA), is a global health concern (1, 2). There is an urgent need to develop novel antimicrobial agents to treat serious life-threatening bacterial infections, especially due to CRE and CRAB as critical priority pathogens (3, 4).

Polymyxins and aminoglycosides are used to treat CR-GNB infections, but their use is hampered by issues of nephrotoxicity, increasing rates of resistance, and insufficient lung concentrations, while tetracyclines, such as tigecycline and eravacycline, have typically bacteriostatic activity rather than bactericidal activity and issues of poor exposure in the lung and urinary tract, respectively, at the clinical dose (5, 6).

The main carbapenem resistance mechanism is the production of carbapenemases. These degrade carbapenem antibiotics and are divided into two groups according to active site: serine-carbapenemases and metallo-carbapenemases (7). One strategy to overcome CR-GNB infection is to combine a β-lactam with a carbapenemase inhibitor to protect the β-lactam from carbapenemase hydrolysis (7, 8). New β-lactamase inhibitors (BLIs) such as avibactam, vaborbactam, and relebactam inhibit serine-carbapenemases from *Enterobacterales* but have no inhibitory activity against oxacillinase (OXA) carbapenemases from *Acinetobacter* spp. and metallo-carbapenemases. Sulbactam/durlobactam is a novel β-lactam/BLI for the treatment of CRAB infections; however, it has limited activity against CR-GNBs (9). Taniborbactam and xeruborbactam are boronic acid-based BLIs currently under development in combination with cefepime and meropenem (MEM), respectively. They inhibit serine- and metallo-carbapenemases, but the former cannot inhibit imipenemases (IMPs) and OXA carbapenemases from *Acinetobacter* spp. while the latter has a high protein binding rate of over 90% in humans (10, 11). Cefiderocol is one of the few viable β-lactam therapy treatment options for infections caused by CR-GNB, especially those involving metallo-carbapenemase producers. However, a recent clinical study reported a higher mortality rate in the subset of patients with CRAB who received cefiderocol (12).

**Fig 1:**
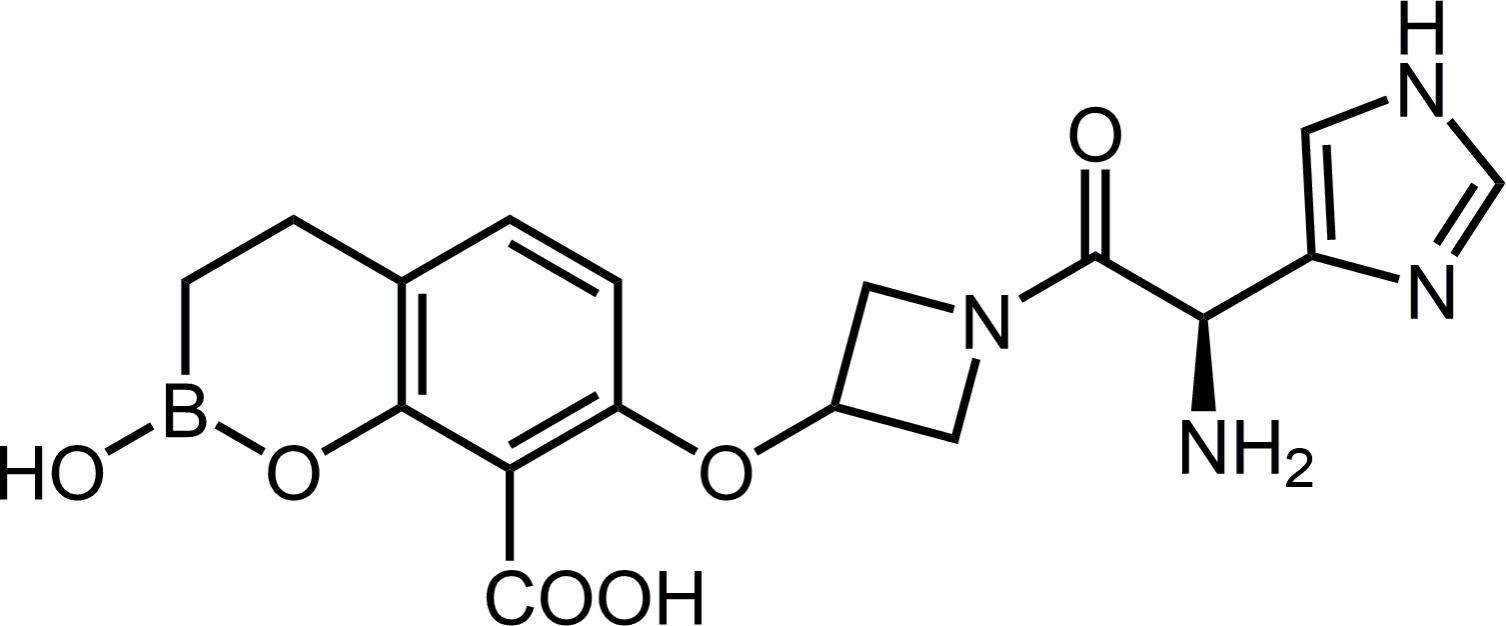
Chemical structure of KSP-1007.

While evaluating new treatments for CR-GNB infections using MEM, which we are the originator of, we discovered a new broad-spectrum boronic acid BLI, KSP-1007, 7-({1-[(2*R*)-2-amino-2-(1*H*-imidazol-4-yl)acetyl]azetidin-3-yl}oxy)-2-hydroxy-3,4-dihydro-2*H*-1,2-benzoxaborinine-8-carboxylic acid (Fig. 1). In this study, we investigated the *in vitro* activities of KSP-1007 in combination with MEM against *Enterobacterales*, *A. baumannii*, and *P. aeruginosa* including carbapenem-resistant and carbapenemase-producing isolates, compared with those of comparators, e.g., MEM/vaborbactam, imipenem/relebactam, ceftazidime/avibactam, aztreonam/avibactam, cefepime/taniborbactam, colistin, tigecycline, and cefiderocol. We compared the effects of human serum albumin on the *in vitro* activities of KSP-1007 and xeruborbactam in combination with MEM against carbapenemase-producing *Enterobacterales* (CPE) and carbapenemase-producing *A. baumannii* (CPAB). The biochemical characterization of KSP-1007 for key serine- and metallo-carbapenemases, *Klebsiella pneumoniae* carbapenemases (KPCs), OXAs, New Delhi metallo-β-lactamases (NDMs), Verona integron-encoded metallo-β-lactamases (VIMs), and IMPs, was also described. We evaluated the therapeutic efficacy of subcutaneous MEM/KSP-1007 administration in several CRE, CRAB, and CRPA systemic murine infection models, neutropenic mouse models of complicated urinary tract infection due to CRE, and CRAB thigh infection models.

## RESULTS

### Biochemical characteristics against various β-lactamases

KSP-1007 strongly inhibited class A carbapenemase KPCs with apparent inhibition constant (*K_i_*_app_) values in the range of 0.836 to 2.19 nM, with a similar potency to avibactam, relebactam, and taniborbactam and superior to the other comparators with *K_i_*_app_ values from 48.3 to 270 nM (Table 1). KSP-1007 was a potent inhibitor of class D carbapenemases, OXAs from *Acinetobacter* spp., and OXA-48 with *K_i_*_app_ of 0.621 to 5.78 nM, which was much lower than those of all the comparators. KSP-1007 also inhibited class B metallo-β-lactamases (MBLs) with *K_i_*_app_ values ranging from 31.6 nM to 1.21 µM (except for IMP-6), whereas none of the comparators, except for taniborbactam, had any MBL-inhibitory activity. The inhibitory activities of KSP-1007 against NDMs and IMPs were superior to those of taniborbactam.

**TABLE 1:** *K*_*i* app_ values of KSP-1007 and comparators with various β-lactamases^*a*^^,^^*c*^

Ambler class	β-Lactamase	*K*_*i* app_ (nM)					
		KSP-1007	Tanibobactam	Vaborbactam	Avibactam	Relebactam	Tazobactam
Class A	CTX-M-14	0.153 ± 0.0235	0.0607 ± 0.0257	392 ± 20.3	2.35 ± 0.763	5.96 ± 3.22	0.0793 ± 0.0256
Class A	CTX-M-15	0.0664 ± 0.015	0.0245 ± 0.0103	78.1 ± 6.63	0.369 ± 0.0569	0.841 ± 0.235	0.0387 ± 0.00434
Class A	CTX-M-27	0.159 ± 0.0228	0.0344 ± 0.0130	351 ± 28.7	1.29 ± 0.663	3.62 ± 1.20	0.0403 ± 0.0162
Class A	SHV-2	361 ± 127	20.0 ± 2.39	n.d.	2.26 ± 1.44	0.373 ± 0.0856	0.187 ± 0.104
Class A	SHV-5	127 ± 16.0	5.90 ± 1.80	1,420 ± 37.5	2.15 ± 0.264	6.26 ± 1.53	1.84 ± 0.243
Class A	SHV-12	53.5 ± 12.2	4.73 ± 0.182	755 ± 103	2.55 ± 0.696	19.3 ± 6.32	1.68 ± 0.266
Class A	TEM-10	6.90 ± 0.879	27.3 ± 4.53	219 ± 67.8	0.230 ± 0.0587	6.83 ± 3.48	0.938 ± 0.149
Cass C	ACT-17	38.3 ± 6.96	12.6 ± 1.55	427 ± 39.3	0.980 ± 0.162	0.498 ± 0.0114	12.1 ± 3.03
Class C	PDC-11	0.790 ± 0.0379	34.6 ± 2.20	3,860 ± 163	107 ± 20.2	48.0 ± 11.9	40.5 ± 20.0
Class A	KPC-2	1.46 ± 0.0574	1.10 ± 0.229	63.7 ± 24.9	3.96 ± 2.16	5.03 ± 0.734	159 ± 48.2
Class A	KPC-3	2.19 ± 0.687	0.947 ± 0.259	88.3 ± 30.3	5.70 ± 1.19	7.67 ± 1.64	270 ± 80.8
Class A	KPC-38	0.836 ± 0.414	0.617 ± 0.0841	48.3 ± 13.2	1.66 ± 0.329	2.94 ± 0.982	112 ± 14.5
Class D	OXA-48	0.861 ± 0.0767	204 ± 31.0	n.d.	2.41 ± 1.62	89.3 ± 46.7	5.32 ± 0.329
Class D	OXA-23	4.07 ± 0.237	n.d.	n.d.	161 ± 36.3	5,640 ± 522	14.0 ± 5.31
Class D	OXA-24	1.09 ± 0.387	2,080 ± 171	n.d.	112 ± 71.2	n.d.	100 ± 45.6
Class D	OXA-65	5.44 ± 1.21	n.d.	n.d.	5,900 ± 1,050	n.d.	n.d.
Class D	OXA-66	5.78 ± 0.654	61,700 ± 2,830	n.d.	7,350 ± 963	n.d.	n.d.
Class D	OXA-72	0.621 ± 0.0248	25,200 ± 1,670	n.d.	691 ± 63.8	≥37,400^*b*^	78.9 ± 8.96
Class B	VIM-1	350 ± 45.4	271 ± 46.7	n.d.	n.d.	n.d.	n.d.
Class B	VIM-2	1,210 ± 337	322 ± 50.0	n.d.	n.d.	n.d.	n.d.
Class B	IMP-1	512 ± 119	n.d.	n.d.	n.d.	n.d.	n.d.
Class B	IMP-6	19,900 ± 3,170	n.d.	n.d.	n.d.	n.d.	n.d.
Class B	NDM-1	126 ± 58.1	2,660 ± 381	n.d.	n.d.	n.d.	n.d.
Class B	NDM-4	153 ± 45.2	1,810 ± 228	n.d.	n.d.	n.d.	n.d.
Class B	NDM-5	31.6 ± 12.3	393 ± 46.4	n.d.	n.d.	n.d.	n.d.
Class B	NDM-6	1,050 ± 37.5	5,290 ± 168	n.d.	n.d.	n.d.	n.d.
Class B	NDM-7	53.0 ± 30.2	704 ± 114	n.d.	n.d.	n.d.	n.d.

^*a*^ *K*_*i* app_ values are expressed as means obtained from three independent studies.

^*b*^ *K_i_*_app_ value was not determined in two out of three independent studies*.*

^*c*^ n.d., not determined due to low inhibitory activity at 100 μM.

Among class A extended-spectrum β-lactamases and class C cephalosporinases, KSP-1007 yielded *K_i_*_app_ values of 0.0664 to 0.790 nM for the inhibition of CTX-Ms and PDC-11, lower than those of all comparators, except for tazobactam and taniborbactam against CTX-Ms. The *K_i_*_app_ values of KSP-1007 against SHV-2, SHV-5, SHV-12, TEM-10, and ACT-17 were 361, 127, 53.5, 6.90, and 38.3 nM, respectively, and were higher than those of avibactam, relebactam, tazobactam, and taniborbactam. Collectively, KSP-1007 was the only compound that inhibited all β-lactamases from classes A, B, C, and D.

### *In vitro* antimicrobial activity

The MIC_50_ and MIC_90_ of MEM against 207 *Enterobacterales* including carbapenem-resistant isolates were 4 and 32 µg/mL, respectively (Table 2). KSP-1007 tested at 4 to 16 µg/mL restored the activity of MEM, with MIC_50_ and MIC_90_ decreasing to ≤0.06 and 8 µg/mL for the combination of MEM/KSP-1007 (4 µg/mL), ≤0.06 and 1 µg/mL for the combination of MEM/KSP-1007 (8 µg/mL), and ≤0.06 and 0.25 µg/mL for the combination of MEM/KSP-1007 (16 µg/mL), respectively. The addition of more than 8 µg/mL KSP-1007 lowered the MIC_90_ of MEM at or below the Clinical and Laboratory Standards Institute (CLSI)-susceptible breakpoint for MEM alone (1 µg/mL), which was lower than those of almost all comparators including MEM/vaborbactam, imipenem/relebactam, ceftolozane/tazobactam, aztreonam/avibactam, and cefiderocol. Additionally, except for cefiderocol, tigecycline (FDA-susceptible breakpoint), and aztreonam/avibactam (based on CLSI breakpoints for aztreonam), the MIC_90_s of comparators were above their susceptible breakpoints. For 127 or 92 MEM-nonsusceptible *Enterobacterales*, the MIC_90_s of MEM/KSP-1007 were 2 to 4 times higher than those for 207 or 154 *Enterobacterales* and were equivalent to those of aztreonam/avibactam, cefiderocol, and tigecycline at ≥8 µg/mL of KSP-1007. The fixed concentration of KSP-1007 at which the MIC_90_ was below the CLSI-susceptible breakpoint for MEM alone was 16 µg/mL.

**TABLE 2:** Antimicrobial activities of MEM in combination with KSP-1007 against GNB including carbapenemase producers^*a*^

Organism (no. tested) and antimicrobial agent (fixed concentration, μg/mL)	MIC (μg/mL)			CLSI MIC interpretation (%)^*b*^		
	Range	MIC_50_	MIC_90_	S	I	R
**All** ***Enterobacterales*** **(207)**						
MEM	≤0.06–>32	4	32	38.6	11.1	50.2
MEM/KSP-1007 (4)	≤0.06–>32	≤0.06	8	86.0^*c*^	1.9^*c*^	12.1^*c*^
MEM/KSP-1007 (8)	≤0.06–>32	≤0.06	1	90.8^*c*^	2.4^*c*^	6.8^*c*^
MEM/KSP-1007 (16)	≤0.06–>32	≤0.06	0.25	96.1^*c*^	2.4^*c*^	1.4^*c*^
MEM/vaborbactam (8)	≤0.06–>32	0.25	32	75.8	6.8	17.4
Ceftazidime	≤0.06–>32	>32	>32	15.5	2.4	82.1
Ceftazidime/avibactam (4)	≤0.06–>32	2	>32	62.8	–	37.2
Imipenem	≤0.06–>32	4	32	36.7	7.7	55.6
Imipenem/relebactam (4)	≤0.06–>32	0.5	16	61.4	7.7	30.9
Ceftolozane/tazobactam (4)	0.25–>64	>64	>64	15.9	2.9	81.2
Cefiderocol^*e*^	≤0.06–16	0.5	2	97.6	2	0.5
Piperacillin/tazobactam (4)	0.25–>64	>64	>64	20.3	3.9	75.8
Tigecycline	0.12–16	0.5	2	95.2^*d*^	4.3^*d*^	0.5^*d*^
Colistin	0.25–>64	0.5	>64	–	78.3	21.7
Amikacin	0.25–>64	4	>64	57	6.3	36.7
Ciprofloxacin	≤0.06–>64	64	>64	23.2	2.4	74.4
Levofloxacin	≤0.06–>64	16	64	24.2	3.9	72
**MEM-nonsusceptible** ***Enterobacterales*** **(127)**						
MEM	2–>32	8	>32	0	18.1	81.9
MEM/KSP-1007 (4)	≤0.06–>32	≤0.06	16	77.2^*c*^	3.1^*c*^	19.7^*c*^
MEM/KSP-1007 (8)	≤0.06–>32	≤0.06	4	85.0^*c*^	3.9^*c*^	11.0^*c*^
MEM/KSP-1007 (16)	≤0.06–>32	≤0.06	1	93.7^*c*^	3.9^*c*^	2.4^*c*^
MEM/vaborbactam (8)	≤0.06–>32	2	32	60.6	11	28.3
Ceftazidime	≤0.06–>32	>32	>32	9.4	3.1	87.4
Ceftazidime/avibactam (4)	≤0.06–>32	2	>32	55.1	–	44.9
Imipenem	0.25–>32	8	>32	10.2	10.2	79.5
Imipenem/relebactam (4)	≤0.06–>32	2	32	48	7.9	44.1
Ceftolozane/tazobactam (4)	0.5–>64	>64	>64	7.1	2.4	90.6
Cefiderocol^*f*^	≤0.06–16	1	2	96	3.2	0.8
Piperacillin/tazobactam (4)	1–>64	>64	>64	7.1	0.8	92.1
Tigecycline	0.12–16	1	2	93.7^*d*^	5.5^*d*^	0.8^*d*^
Colistin	0.25–>64	0.5	>64	–	77.2	22.8
Amikacin	0.5–>64	16	>64	40.2	7.9	52
Ciprofloxacin	≤0.06–>64	64	>64	16.5	2.4	81.1
Levofloxacin	≤0.06–>64	32	64	18.1	2.4	79.5
***Enterobacterales*** **(154)**^*g*^						
MEM	≤0.06–>32	2	32	40.3	9.7	50
MEM/KSP-1007 (4)	≤0.06–>32	≤0.06	4	87.0^*c*^	1.9^*c*^	11.0^*c*^
MEM/KSP-1007 (8)	≤0.06–>32	≤0.06	1	92.9^*c*^	2.6^*c*^	4.5^*c*^
MEM/KSP-1007 (16)	≤0.06–>32	≤0.06	0.25	98.7^*c*^	0.6^*c*^	0.6^*c*^
Aztreonam	≤0.03–>64	>64	>64	16.9	2.6	80.5
Aztreonam/avibactam (4)	≤0.03–8	0.12	1	99.4^*c*^	0.6^*c*^	0.0^*c*^
**MEM-nonsusceptible** ***Enterobacterales*** **(92)**^*g*^						
MEM	2–>32	8	>32	0	16.3	83.7
MEM/KSP-1007 (4)	≤0.06–>32	≤0.06	16	78.3^*c*^	3.3^*c*^	18.5^*c*^
MEM/KSP-1007 (8)	≤0.06–>32	≤0.06	2	88.0^*c*^	4.3^*c*^	7.6^*c*^
MEM/KSP-1007 (16)	≤0.06–>32	≤0.06	0.5	97.8^*c*^	1.1^*c*^	1.1^*c*^
Aztreonam	≤0.03–>64	>64	>64	8.7	1.1	90.2
Aztreonam/avibactam (4)	≤0.03–4	0.12	1	100^*c*^	0.0^*c*^	0.0^*c*^
***Enterobacterales*** **with serine-carbapenemases (80)**						
MEM	≤0.06–>32	4	32	27.5	11.3	61.3
MEM/KSP-1007 (4)	≤0.06–>32	≤0.06	0.12	95.0^*c*^	0.0^*c*^	5.0^*c*^
MEM/KSP-1007 (8)	≤0.06–16	≤0.06	≤0.06	98.8^*c*^	0.0^*c*^	1.3^*c*^
MEM/KSP-1007 (16)	≤0.06–0.5	≤0.06	≤0.06	100^*c*^	0.0^*c*^	0.0^*c*^
MEM/vaborbactam (8)	≤0.06–32	≤0.06	1	95	3.8	1.3
Ceftazidime	≤0.06–>32	>32	>32	23.8	2.5	73.8
Ceftazidime/avibactam (4)	≤0.06–>32	0.5	2	97.5	–	2.5
Imipenem	0.5–>32	8	>32	23.8	7.5	68.8
Imipenem/relebactam (4)	≤0.06–4	0.25	2	82.5	13.8	3.8
Ceftolozane/tazobactam (4)	0.25–>64	64	>64	18.8	1.3	80
Cefiderocol	≤0.06–4	0.25	2	100	0	0
Piperacillin/tazobactam (4)	0.25–>64	>64	>64	15	1.3	83.8
Tigecycline	0.12–4	1	2	96.3^*d*^	3.8^*d*^	0.0^*d*^
Colistin	0.25–>64	0.5	>64	–	73.8	26.3
Amikacin	0.5–>64	4	64	53.8	8.8	37.5
Ciprofloxacin	≤0.06–>64	32	>64	26.3	0	73.8
Levofloxacin	≤0.06–>64	16	>64	27.5	1.3	71.3
**MEM-nonsusceptible** ***Enterobacterales*** **with serine-carbapenemases (58)**						
MEM	2–>32	8	32	0	15.5	84.5
MEM/KSP-1007 (4)	≤0.06–>32	≤0.06	1	93.1^*c*^	0.0^*c*^	6.9^*c*^
MEM/KSP-1007 (8)	≤0.06–16	≤0.06	0.12	98.3^*c*^	0.0^*c*^	1.7^*c*^
MEM/KSP-1007 (16)	≤0.06–0.5	≤0.06	0.12	100^*c*^	0.0^*c*^	0.0^*c*^
MEM/vaborbactam (8)	≤0.06–32	≤0.06	1	93.1	5.2	1.7
Ceftazidime	0.12–>32	>32	>32	15.5	1.7	82.8
Ceftazidime/avibactam (4)	≤0.06–>32	1	4	96.6	–	3.4
Imipenem	1–>32	8	>32	5.2	10.3	84.5
Imipenem/relebactam (4)	≤0.06–4	0.25	2	84.5	12.1	3.4
Ceftolozane/tazobactam (4)	0.5–>64	64	>64	15.5	1.7	82.8
Cefiderocol	≤0.06–4	0.5	2	100	0	0
Piperacillin/tazobactam (4)	1–>64	>64	>64	13.8	0	86.2
Tigecycline	0.12–4	1	2	98.3^*d*^	1.7^*d*^	0.0^*d*^
Colistin	0.25–>64	0.5	>64	–	74.1	25.9
Amikacin	0.5–>64	8	64	44.8	10.3	44.8
Ciprofloxacin	≤0.06–>64	64	>64	20.7	0	79.3
Levofloxacin	≤0.06–>64	32	64	24.1	0	75.9
***Enterobacterales*** **with serine-carbapenemases (61)**^*g*^						
MEM	0.12–>32	4	32	31.1	11.5	57.4
MEM/KSP-1007 (4)	≤0.06–>32	≤0.06	≤0.06	95.1^*c*^	0.0^*c*^	4.9^*c*^
MEM/KSP-1007 (8)	≤0.06–16	≤0.06	≤0.06	98.4^*c*^	0.0^*c*^	1.6^*c*^
MEM/KSP-1007 (16)	≤0.06–0.5	≤0.06	≤0.06	100^*c*^	0.0^*c*^	0.0^*c*^
Aztreonam	≤0.03–>64	>64	>64	11.5	3.3	85.2
Aztreonam/avibactam (4)	≤0.03–1	0.12	0.25	100^*c*^	0.0^*c*^	0.0^*c*^
**MEM-nonsusceptible** ***Enterobacterales*** **with serine-carbapenemases (42)**^*g*^						
MEM	2–>32	8	32	0	16.7	83.3
MEM/KSP-1007 (4)	≤0.06–>32	≤0.06	1	92.9^*c*^	0.0^*c*^	7.1^*c*^
MEM/KSP-1007 (8)	≤0.06–16	≤0.06	≤0.06	97.6^*c*^	0.0^*c*^	2.4^*c*^
MEM/KSP-1007 (16)	≤0.06–0.5	≤0.06	≤0.06	100^*c*^	0.0^*c*^	0.0^*c*^
Aztreonam	0.12–>64	>64	>64	4.8	2.4	92.9
Aztreonam/avibactam (4)	≤0.03–1	0.12	0.25	100^*c*^	0.0^*c*^	0.0^*c*^
***Enterobacterales*** **with metallo-carbapenemases (74)**						
MEM	0.12–>32	8	>32	25.7	9.5	64.9
MEM/KSP-1007 (4)	≤0.06–>32	≤0.06	32	78.4^*c*^	1.4^*c*^	20.3^*c*^
MEM/KSP-1007 (8)	≤0.06–>32	≤0.06	8	82.4^*c*^	2.7^*c*^	14.9^*c*^
MEM/KSP-1007 (16)	≤0.06–>32	≤0.06	1	91.9^*c*^	4.1^*c*^	4.1^*c*^
MEM/vaborbactam (8)	≤0.06–>32	8	>32	40.5	13.5	45.9
Ceftazidime	≤0.06–>32	>32	>32	1.4	0	98.6
Ceftazidime/avibactam (4)	0.5–>32	>32	>32	1.4	–	98.6
Imipenem	0.12–>32	4	>32	20.3	9.5	70.3
Imipenem/relebactam (4)	0.12–>32	8	>32	17.6	4.1	78.4
Ceftolozane/tazobactam (4)	2–>64	>64	>64	1.4	0	98.6
Cefiderocol*^h^*	≤0.06–16	1	4	93.2	5.5	1.4
Piperacillin/tazobactam (4)	2–>64	>64	>64	14.9	5.4	79.7
Tigecycline	0.12–4	0.5	2	91.9^*d*^	8.1^*d*^	0.0^*d*^
Colistin	0.25–>64	0.5	4	–	89.2	10.8
Amikacin	0.5–>64	16	>64	41.9	5.4	52.7
Ciprofloxacin	≤0.06–>64	64	>64	12.2	5.4	82.4
Levofloxacin	≤0.06–>64	32	>64	12.2	6.8	81.1
**MEM-nonsusceptible** ***Enterobacterales*** **with metallo-carbapenemases (55)**						
MEM	2–>32	16	>32	0	12.7	87.3
MEM/KSP-1007 (4)	≤0.06–>32	0.12	>32	70.9^*c*^	1.8^*c*^	27.3^*c*^
MEM/KSP-1007 (8)	≤0.06–>32	≤0.06	8	76.4^*c*^	3.6^*c*^	20.0^*c*^
MEM/KSP-1007 (16)	≤0.06–>32	≤0.06	2	89.1^*c*^	5.5^*c*^	5.5^*c*^
MEM/vaborbactam (8)	1–>32	16	>32	20	18.2	61.8
Ceftazidime	≤0.06–>32	>32	>32	1.8	0	98.2
Ceftazidime/avibactam (4)	16–>32	>32	>32	0	–	100
Imipenem	0.5–>32	16	>32	9.1	9.1	81.8
Imipenem/relebactam (4)	0.5–>32	16	>32	3.6	1.8	94.5
Ceftolozane/tazobactam (4)	32–>64	>64	>64	0	0	100
Cefiderocol^*i*^	≤0.06–16	1	4	90.7	7.4	1.9
Piperacillin/tazobactam (4)	8–>64	>64	>64	1.8	1.8	96.4
Tigecycline	0.12–4	1	4	89.1^*d*^	10.9^*d*^	0.0^*d*^
Colistin	0.25–>64	0.5	8	–	85.5	14.5
Amikacin	0.5–>64	32	>64	29.1	5.5	65.5
Ciprofloxacin	≤0.06–>64	64	>64	5.5	5.5	89.1
Levofloxacin	≤0.06–>64	32	>64	5.5	5.5	89.1
***Enterobacterales*** **with metallo-carbapenemases (47)**^*g*^						
MEM	0.12–>32	16	>32	12.8	10.6	76.6
MEM/KSP-1007 (4)	≤0.06–>32	≤0.06	32	78.7^*c*^	2.1^*c*^	19.1^*c*^
MEM/KSP-1007 (8)	≤0.06–>32	≤0.06	8	85.1^*c*^	4.3^*c*^	10.6^*c*^
MEM/KSP-1007 (16)	≤0.06–>32	≤0.06	1	95.7^*c*^	2.1^*c*^	2.1^*c*^
MEM/taniborbactam (4)	≤0.06–>32	0.5	>32	70.2^*c*^	2.1^*c*^	27.7^*c*^
Cefepime	2–>64	>64	>64	4.3	2.1	93.6
Cefepime/taniborbactam (4)	≤0.06–>64	8	64	46.8^*c*^	19.1^*c*^	34.0^*c*^
Cefepime/KSP-1007 (4)	≤0.06–>64	1	>64	66.0^*c*^	6.4^*c*^	27.7^*c*^
Aztreonam	≤0.03–>64	>64	>64	19.1	0	80.9
Aztreonam/avibactam (4)	≤0.03–4	0.12	2	100^*c*^	0.0^*c*^	0.0^*c*^
**MEM-nonsusceptible** ***Enterobacterales*** **with metallo-carbapenemases (41)**^*g*^						
MEM	2–>32	16	>32	0	12.2	87.8
MEM/KSP-1007 (4)	≤0.06–>32	≤0.06	32	75.6^*c*^	2.4^*c*^	22.0^*c*^
MEM/KSP-1007 (8)	≤0.06–>32	≤0.06	8	82.9^*c*^	4.9^*c*^	12.2^*c*^
MEM/KSP-1007 (16)	≤0.06–>32	≤0.06	1	95.1^*c*^	2.4^*c*^	2.4^*c*^
MEM/taniborbactam (4)	≤0.06–>32	0.5	>32	68.3^*c*^	2.4^*c*^	29.3^*c*^
Cefepime	2–>64	>64	>64	2.4	0	97.6
Cefepime/taniborbactam (4)	≤0.06–>64	8	64	41.5^*c*^	19.5^*c*^	39.0^*c*^
Cefepime/KSP-1007 (4)	≤0.06–>64	2	>64	63.4^*c*^	4.9^*c*^	31.7^*c*^
Aztreonam	≤0.03–>64	>64	>64	14.6	0	85.4
Aztreonam/avibactam (4)	≤0.03–4	0.25	2	100^*c*^	0.0^*c*^	0.0^*c*^
**Cefepime/taniborbactam-nonsusceptible** ***Enterobacterales*** **with metallo-carbapenemases (25)**^*c*^^,^^*g*^						
MEM	1–>32	32	>32	4	0	96
MEM/KSP-1007 (4)	≤0.06–>32	0.12	>32	60.0^*c*^	4.0^*c*^	36.0^*c*^
MEM/KSP-1007 (8)	≤0.06–>32	0.12	32	72.0^*c*^	8.0^*c*^	20.0^*c*^
MEM/KSP-1007 (16)	≤0.06–>32	≤0.06	1	92.0^*c*^	4.0^*c*^	4.0^*c*^
MEM/taniborbactam (4)	0.12–>32	4	>32	44.0^*c*^	4.0^*c*^	52.0^*c*^
Cefepime	8–>64	>64	>64	0	4	96
Cefepime/taniborbactam (4)	4–>64	16	64	0.0^*c*^	36.0^*c*^	64.0^*c*^
Cefepime/KSP-1007 (4)	0.25–>64	16	>64	40.0^*c*^	8.0^*c*^	52.0^*c*^
Aztreonam	1–>64	>64	>64	4	0	96
Aztreonam/avibactam (4)	≤0.03–4	0.5	2	100^*c*^	0.0^*c*^	0.0^*c*^
***Acinetobacter baumannii*** **(55)**						
MEM	2–>128	32	>128	5.5	5.5	89.1
MEM/KSP-1007 (4)	1–128	4	32	21.8^*c*^	29.1^*c*^	49.1^*c*^
MEM/KSP-1007 (8)	1–32	4	8	49.1^*c*^	38.2^*c*^	12.7^*c*^
MEM/KSP-1007 (16)	≤0.25–4	2	4	89.1^*c*^	10.9^*c*^	0.0^*c*^
MEM/vaborbactam (8)	2–>128	32	>128	5.5^*c*^	5.5^*c*^	89.1^*c*^
Ceftazidime	8–>32	>32	>32	3.6	1.8	94.5
Ceftazidime/avibactam (4)	4–>32	32	>32	7.3^*c*^	14.5^*c*^	78.2^*c*^
Imipenem	1–>32	32	>32	9.1	3.6	87.3
Imipenem/relebactam (4)	0.5–>32	32	>32	9.1^*c*^	3.6^*c*^	87.3^*c*^
Ceftolozane/tazobactam (4)	2–>64	32	>64	–	–	–
Cefiderocol	≤0.06–16	0.5	4	94.5	3.6	1.8
Piperacillin/tazobactam (4)	64–>64	>64	>64	0	1.8	98.2
Aztreonam	32–>64	64	>64	–	–	–
Aztreonam/avibactam (4)	16–>64	64	>64	–	–	–
Tigecycline	0.25–2	1	1	–	–	–
Colistin	0.25–>64	1	2	–	94.5	5.5
Amikacin	0.5–>64	64	>64	29.1	14.5	56.4
Ciprofloxacin	8–>64	64	>64	0	0	100
Levofloxacin	4–>64	16	64	0	10.9	89.1
***Acinetobacter baumannii*** **(46)**						
MEM	16–>128	32	>128	0	0	100
MEM/KSP-1007 (4)	1–128	8	64	13.0^*c*^	30.4^*c*^	56.5^*c*^
MEM/KSP-1007 (8)	1–32	4	8	43.5^*c*^	41.3^*c*^	15.2^*c*^
MEM/KSP-1007 (16)	0.5–4	2	2	91.3^*c*^	8.7^*c*^	0.0^*c*^
MEM/taniborbactam (4)	16–>128	64	>128	0.0^*c*^	0.0^*c*^	100^*c*^
Cefepime	16–>256	64	>256	0	4.3	95.7
Cefepime/taniborbactam (4)	8–>256	32	128	2.2^*c*^	15.2^*c*^	82.6^*c*^
Cefepime/KSP-1007 (4)	8–256	32	128	6.5^*c*^	30.4^*c*^	63.0^*c*^
***Pseudomonas aeruginosa*** **(96)**						
MEM	≤0.25–>128	16	>128	20.8	11.5	67.7
MEM/KSP-1007 (4)	≤0.25–>128	8	128	25.0^*c*^	11.5^*c*^	63.5^*c*^
MEM/KSP-1007 (8)	≤0.25–>128	8	128	28.1^*c*^	10.4^*c*^	61.5^*c*^
MEM/KSP-1007 (16)	≤0.25–>128	8	64	35.4^*c*^	12.5^*c*^	52.1^*c*^
MEM/vaborbactam (8)	≤0.25–>128	8	>128	22.9^*c*^	9.4^*c*^	67.7^*c*^
Ceftazidime	1–>32	32	>32	37.5	6.3	56.3
Ceftazidime/avibactam (4)	0.5–>32	4	>32	61.5	–	38.5
Imipenem	0.25–>32	16	>32	13.5	3.1	83.3
Imipenem/relebactam (4)	0.25–>32	2	>32	55.2	12.5	32.3
Ceftolozane/tazobactam (4)	0.5–>64	4	>64	53.1	2.1	44.8
Cefiderocol	≤0.06–4	0.5	2	100	0	0
Piperacillin/tazobactam (4)	0.5–>64	64	>64	35.4	4.2	60.4
Aztreonam	0.5–>64	16	>64	39.6	12.5	47.9
Aztreonam/avibactam (4)	0.12–>64	8	32	55.2^*c*^	21.9^*c*^	22.9^*c*^
Tigecycline	0.5–16	4	16	–	–	–
Colistin	0.25–2	1	1	–	100	0
Amikacin	0.25–>64	8	>64	63.5	7.3	29.2
Ciprofloxacin	0.12–>64	16	64	17.7	4.2	78.1
Levofloxacin	0.5–>64	32	64	14.6	4.2	81.3
**MEM-nonsusceptible** ***Pseudomonas aeruginosa*** **(76)**						
MEM	4–>128	16	>128	0	14.5	85.5
MEM/KSP-1007 (4)	2–>128	16	>128	5.3^*c*^	14.5^*c*^	80.3^*c*^
MEM/KSP-1007 (8)	0.5–>128	16	>128	10.5^*c*^	11.8^*c*^	77.6^*c*^
MEM/KSP-1007 (16)	≤0.25–>128	8	128	18.4^*c*^	15.8^*c*^	65.8^*c*^
MEM/vaborbactam (8)	2–>128	16	>128	3.9^*c*^	10.5^*c*^	85.5^*c*^
Ceftazidime	1–>32	32	>32	25	6.6	68.4
Ceftazidime/avibactam (4)	1–>32	8	>32	51.3	–	48.7
Imipenem	1–>32	32	>32	3.9	2.6	93.4
Imipenem/relebactam (4)	0.5–>32	4	>32	43.4	15.8	40.8
Ceftolozane/tazobactam (4)	0.5–>64	16	>64	42.1	2.6	55.3
Cefiderocol	≤0.06–4	0.5	2	100	0	0
Piperacillin/tazobactam (4)	2–>64	>64	>64	21.1	5.3	73.7
Aztreonam	2–>64	32	>64	30.3	13.2	56.6
Aztreonam/avibactam (4)	2–>64	16	32	46.1^*c*^	26.3^*c*^	27.6^*c*^
Tigecycline	1–16	4	16	–	–	–
Colistin	0.25–2	1	1	–	100	0
Amikacin	0.25–>64	8	>64	55.3	7.9	36.8
Ciprofloxacin	0.12–>64	16	64	10.5	3.9	85.5
Levofloxacin	0.5–>64	32	>64	9.2	1.3	89.5
**Imipenem/relebactam-nonsusceptible** ***Pseudomonas aeruginosa*** **(43)**						
MEM	4–>128	64	>128	0	4.7	95.3
MEM/KSP-1007 (4)	2–>128	64	>128	4.7^*c*^	4.7^*c*^	90.7^*c*^
MEM/KSP-1007 (8)	1–>128	32	>128	7.0^*c*^	2.3^*c*^	90.7^*c*^
MEM/KSP-1007 (16)	≤0.25–>128	16	>128	9.3^*c*^	2.3^*c*^	88.4^*c*^
MEM/vaborbactam (8)	4–>128	64	>128	0.0^*c*^	2.3^*c*^	97.7^*c*^
Ceftazidime	4–>32	>32	>32	9.3	7	83.7
Ceftazidime/avibactam (4)	4–>32	32	>32	20.9	–	79.1
Imipenem	2–>32	>32	>32	2.3	0	97.7
Imipenem/relebactam (4)	4–>32	>32	>32	0	27.9	72.1
Ceftolozane/tazobactam (4)	1–>64	>64	>64	14	2.3	83.7
Cefiderocol	0.12–4	0.5	2	100	0	0
Piperacillin/tazobactam (4)	4–>64	>64	>64	7	4.7	88.4
Aztreonam	2–>64	32	>64	30.2	9.3	60.5
Aztreonam/avibactam (4)	2–>64	16	64	32.6^*c*^	27.9^*c*^	39.5^*c*^
Tigecycline	1–16	4	16	–	–	–
Colistin	0.5–2	1	1	–	100	0
Amikacin	0.5–>64	64	>64	32.6	9.3	58.1
Ciprofloxacin	1–>64	32	64	0	2.3	97.7
Levofloxacin	4–>64	32	>64	0	0	100
**Ceftolozane/tazobactam-nonsusceptible** ***Pseudomonas aeruginosa*** **(45)**						
MEM	1–>128	64	>128	2.2	6.7	91.1
MEM/KSP-1007 (4)	0.5–>128	32	>128	6.7^*c*^	8.9^*c*^	84.4^*c*^
MEM/KSP-1007 (8)	0.5–>128	16	>128	13.3^*c*^	6.7^*c*^	80.0^*c*^
MEM/KSP-1007 (16)	≤0.25–>128	16	>128	20.0^*c*^	4.4^*c*^	75.6^*c*^
MEM/vaborbactam (8)	1–>128	64	>128	6.7^*c*^	6.7^*c*^	86.7^*c*^
Ceftazidime	8–>32	>32	>32	2.2	4.4	93.3
Ceftazidime/avibactam (4)	1–>32	32	>32	24.4	–	75.6
Imipenem	0.5–>32	>32	>32	4.4	0	95.6
Imipenem/relebactam (4)	0.5–>32	32	>32	17.8	13.3	68.9
Ceftolozane/tazobactam (4)	8–>64	>64	>64	0	4.4	95.6
Cefiderocol	0.25–4	0.5	2	100	0	0
Piperacillin/tazobactam (4)	4–>64	>64	>64	4.4	6.7	88.9
Aztreonam	2–>64	32	>64	28.9	8.9	62.2
Aztreonam/avibactam (4)	2–>64	16	32	40.0^*c*^	31.1^*c*^	28.9^*c*^
Tigecycline	1–16	4	16	–	–	–
Colistin	0.25–2	1	1	–	100	0
Amikacin	0.25–>64	64	>64	33.3	11.1	55.6
Ciprofloxacin	0.12–>64	32	64	8.9	4.4	86.7
Levofloxacin	0.5–>64	32	64	6.7	2.2	91.1

^*a*^ S, susceptible; I, intermediate (including susceptible dose-dependent); R, resistant.

^*b*^ CLSI breakpoints (13).

^*c*^ Based on CLSI breakpoints for MEM, ceftazidime, imipenem, cefepime, or aztreonam (13).

^*d*^ FDA breakpoints for tigecycline (14).

^*e*^ 205 isolates.

^*f*^ 126 isolates.

^*g*^ CDC AR Bank.

^*h*^ 73 isolates.

^*i*^ 54 isolates.

The MIC_90_ of MEM with KSP-1007 at 8 µg/mL against serine-carbapenemase-producing isolates (≤0.12 µg/mL) was lower than that of all comparator agents. When tested against the MBL-producing isolates, only aztreonam/avibactam, cefiderocol, tigecycline, and colistin yielded lower MIC_90_s (≤4 µg/mL) compared to the MIC_90_ of MEM with KSP-1007 at 8 µg/mL. A similar phenomenon was observed for MEM-nonsusceptible carbapenemase-producing isolates. Regardless of MEM- or cefepime/taniborbactam-nonsusceptible isolates, the *in vitro* activity of MEM/KSP-1007 against MBL producers was superior to that of MEM/taniborbactam, cefepime/taniborbactam, and cefepime/KSP-1007 at the same BLI concentration. Comparing KSP-1007 and taniborbactam efficacy against MBL producers under the same β-lactam combination with MEM or cefepime, KSP-1007 was better in terms of susceptibility rate. At a concentration of 16 µg/mL, KSP-1007 in combination with MEM yielded the lowest MIC_90_ value (≤2 µg/mL) among all agents.

Penicillin-binding protein 3 (PBP3) mutations are known to decrease susceptibility to a broad range of β-lactams, except for carbapenems (15). Furthermore, in NDM-producing *Escherichia coli* with these mutations, decreased susceptibility to aztreonam/avibactam, cefepime/taniborbactam, and cefiderocol is reported (15–18). From NDM-producing *E. coli* in CDC isolates, we identified seven isolates harboring PBP3 mutations, with their drug susceptibilities shown in Table 3. MEM/KSP-1007 showed excellent activity against *E. coli* isolates with NDM carbapenemase and PBP3 mutations compared to aztreonam/avibactam, cefepime/taniborbactam, and cefiderocol.

**TABLE 3:** Antimicrobial activities of MEM in combination with KSP-1007 against NDM-producing *E. coli* with PBP3 mutations^*a*^^*, b*^

Organism (carbapenemase)	MIC (μg/mL)						
	MEM	MEM/KSP (4)	FEP	FEP/TAN (4)	ATM	ATM/AVI (4)	FDC
*E. coli* CDC-119 (NDM-1)	32	≤0.06	>64	16	>64	4	0.5
*E. coli* CDC-137 (NDM-6)	32	0.12	>64	8	>64	4	2
*E. coli* CDC-149 (NDM-7)	8	0.5	>64	0.5	0.5	0.12	16
*E. coli* CDC-150 (NDM-5)	>32	≤0.06	>64	16	32	2	4
*E. coli* CDC-151 (NDM-5)	8	≤0.06	>64	16	32	2	4
*E. coli* CDC-162 (NDM-7)	32	≤0.06	>64	16	>64	1	8
*E. coli* CDC-435 (NDM-1)	32	≤0.06	>64	8	>64	2	4

^*a*^ KSP, KSP-1007; FEP, cefepime; TAN, taniborbactam; ATM, aztreonam; AVI, avibactam; FDC, cefiderocol.

^*b*^ Fixed concentration (μg/mL) is shown in parentheses.

KSP-1007 dose-dependently potentiated the activity of MEM against 55 isolates of *A. baumannii* (Table 2). Almost all *A. baumannii* isolates were MEM-nonsusceptible (52/55) and positive for at least one OXA-type serine-carbapenemase (54/55); the NDM-1 MBL was produced in 4/55 isolates (see Table S1 in the supplemental material). The MIC_50_ and MIC_90_ values for MEM decreased from 32 to 2 and from >128 to 4 µg/mL with the addition of KSP-1007 at 4 to 16 µg/mL. The MIC_90_ for MEM with KSP-1007 at 8 µg/mL was lower than those for the other comparators, except for cefiderocol, colistin, and tigecycline. The addition of 8 µg/mL of KSP-1007 reduced the MICs of MEM against four NDM-1-producing isolates (≥128 µg/mL) to the susceptible breakpoint of MEM alone (2 µg/mL). KSP-1007 exhibited superior anti-*Acinetobacter* activity when combined with MEM compared to cefepime. In contrast, taniborbactam, reflecting its inability to inhibit OXA enzymes from *Acinetobacter* spp., did not enhance MEM anti-*Acinetobacter* activity.

MEM MIC_50_ (16 µg/mL) and MIC_90_ (>128 µg/mL) against 96 *P. aeruginosa* were only 2-fold reduced by the addition of KSP-1007 (Table 2). Against MEM-, imipenem/relebactam-, and ceftolozane/tazobactam-nonsusceptible *P. aeruginosa*, KSP-1007 only slightly decreased MEM MIC_50_ or MIC_90_ at 16 µg/mL, the highest concentration tested. These values were higher than those of aztreonam/avibactam, cefiderocol, colistin, and tigecycline. In 16 of 19 isolates harboring VIMs and all 7 isolates harboring serine-type-only carbapenemase, KSP-1007 at 16 µg/mL lowered the MIC of MEM by 4- to 128-fold (see Table S1 in the supplemental material).

### Bactericidal activity

The minimum bactericidal concentration (MBC) of MEM/KSP-1007 (4 µg/mL), MEM/vaborbactam (8 µg/mL), and ceftazidime/avibactam (4 µg/mL) was evaluated against 17 CPE and 4 CPAB. The MICs of MEM/KSP-1007 against CPE and CPAB ranged from 0.015 to 0.25 and from 4 to 8 µg/mL, respectively, and were lower than those of all comparators (Table 4). Almost all the MBC/MIC ratios for MEM alone were ≤4 despite the MBC of more than 32 µg/mL for many strains. MEM/KSP-1007 also demonstrated bactericidal activity against CPE and CPAB given MBC/MIC ratios of ≤4, except for *Enterobacter aerogenes* CDC-161. However, against this strain, the addition of 4 µg/mL KSP-1007 lowered the MBC of MEM to 0.5 µg/mL and below the susceptible breakpoint for MEM alone (1 µg/mL). MEM/KSP-1007 showed more potent *in vitro* bactericidal activity against CPE and CPA used in this study than MEM/vaborbactam and ceftazidime/avibactam. MEM alone and MEM/KSP-1007 (8 µg/mL) demonstrated bactericidal activity against carbapenemase-producing *P. aeruginosa* (CPPA) based on MBC/MIC ratios of ≤4.

**TABLE 4:** MIC and MBC values of MEM in combination with KSP-1007 against carbapenemase-producing *Enterobacterales*, *A. baumannii*, and *P. aeruginosa^a^^, b^*

Organism (no. tested)	Drug	MIC range (µg/mL)	MBC range (µg/mL)	MIC/MBC range
Serine*-*carbapenemase- producing *Enterobacterales* (6)	MEM	1–64	4–128	1–4
	MEM/KSP-1007 (4)	0.015–0.06	0.03–0.06	1–2
	MEM/VAB (8)	0.03–0.5	0.03–4	1–8
	CAZ	0.25–>128	0.5–>128	1–256
	CAZ/AVI (4)	≤0.12–2	≤0.12–2	1–2
Metallo-carbapenemase- producing *Enterobacterales* (11)	MEM	4–128	8–128	1–4
	MEM/KSP-1007 (4)	0.03–0.25	0.03–0.5	1–8
	MEM/VAB (8)	4–128	8–>128	1–≥2
	CAZ	128–>128	128–>128	≥1
	CAZ/AVI (4)	>128	>128	≥1
Carbapenemase-producing *A*. *baumannii* (4)	MEM	64–>128	64–>128	≥1
	MEM/KSP-1007 (4)	4–8	8–16	1–4
	MEM/VAB (8)	64–128	64–>128	1–≥2
	CAZ	64–>128	64–>128	1–2
	CAZ/AVI (4)	16–>128	64–>128	2–4
Carbapenemase-producing *P*. *aeruginosa* (7)	MEM	64–256	64–256	1–2
	MEM/KSP-1007 (8)	1–128	4–128	1–4

^*a*^ VAB, vaborbactam; CAZ, ceftazidime; AVI, avibactam.

^*b*^ Fixed concentrations (μg/mL) are shown in parentheses.

### Effect of human serum albumin on the *in vitro* activity

To compare the effects of human serum albumin (HSA) on the *in vitro* activities of KSP-1007 and xeruborbactam in combination with MEM, MICs were determined using cation-adjusted Mueller Hinton broth (CAMHB), with and without 4% HSA (see Table S3 in the supplemental material). In the presence of 4% HSA, the geometric mean (GM) MIC of MEM/KSP-1007 against 12 strains of CPE was 0.128 µg/mL and remained within 1.5-fold of that in its absence (0.0857 µg/mL). In contrast, MEM/xeruborbactam with 4% HSA yielded a value of 1.32 µg/mL, 14-fold higher than that without HSA (0.0969 µg/mL). Against 12 strains of CPAB, the addition of 4% HSA did not affect the GM MIC of MEM/KSP-1007 (8.48 µg/mL versus 6.35 µg/mL) but increased the value of MEM/xeruborbactam from 1.50 to 12.0 µg/mL (8-fold). Unlike MEM/xeruborbactam, the *in vitro* activity of MEM/KSP-1007 against CPE and CPAB was unaffected by the presence of 4% HSA.

### Intrinsic antibacterial activity

Several diazabicyclooctane BLIs and xeruborbactam have intrinsic antimicrobial activities, thereby exhibiting a β-lactam enhancer effect (19). This prompted our investigation into the intrinsic antimicrobial activity of KSP-1007 against reference bacterial strains. The MICs of KSP-1007 against 12 Gram-positive strains (including methicillin-sensitive staphylococci and streptococci) ranged from 0.25 to 8 µg/mL (see Table S4 in the supplemental material). The MICs of KSP-1007 for the other five Gram-positive strains (including methicillin-resistant staphylococci and enterococci) and all 16 Gram-negative strains (including *Enterobacterales*, *A. baumannii*, and *P. aeruginosa*) were equal to or greater than 64 µg/mL. Thus, the effects of KSP-1007 on the increased MEM efficacy against CR-GNBs observed in Tables 2 and 3 are due to the β-lactamase inhibitory activity of KSP-1007 and might not include the intrinsic antimicrobial activity of KSP-1007.

The MICs of KSP-1007 against 20 anaerobic strains (including *Clostridium perfringens*, *Atopobium rimae*, *Bacteroides* spp., and *Prevotella* spp.) ranged from 2 to 256 µg/mL (see Table S5 in the supplemental material). The MICs for KSP-1007 against the other seven strains were greater than the maximum tested concentration of 256 µg/mL.

### *In vivo* efficacy against CPE, CPAB, and CPPA in murine systemic infection models

In the *in vitro* experiments, the approved BLIs did not inhibit MBL and OXA enzymes from *Acinetobacter* spp.*,* whereas KSP-1007 was effective against these carbapenemases. Next, we confirmed the *in vivo* efficacy of KSP-1007 in combination with MEM in lethal mouse models of systemic infection due to KPC, MBL, and/or OXA enzyme-producing GNB (Table 5).

**TABLE 5:** Activity of MEM alone and in combination with KSP-1007 in lethal mouse models of systemic infection caused by carbapenemase-producing *Enterobacterales*, *A. baumannii*, and *P. aeruginosa^a^*

Organism (carbapenemase) and antimicrobial agent (ratio)	MIC (μg/mL)	ED_50_ of MEM (mg/kg) (95% confidence intervals)
***Klebsiella pneumoniae*** **ATCC BAA-2344 (KPC-2)**		
**Experiment 1**		
MEM	16–32	205 (ND)
MEM/KSP-1007 (4:1)	0.03^*b*^	6.48 (3.78–10.5)
MEM/vaborbactam (4:1)	0.03^*c*^	9.08 (4.40–27.0)
MEM/avibactam (4:1)	0.03^*b*^	6.18 (3.90–9.69)
**Experiment 2**		
MEM	16–32	54.8 (35.5–84.0)
MEM/KSP-1007 (4:1)	0.03^*b*^	5.74 (3.68–8.23)
MEM/taniborbactam (4:1)	0.03^*b*^	6.61 (4.91–8.29)
***E. coli*** **ATCC BAA-2469 (NDM-1)**		
**Experiment 1**		
MEM	32	103 (ND)
MEM/KSP-1007 (4:1)	0.03^*b*^	8.93 (5.90–14.5)
MEM/vaborbactam (4:1)	32^*c*^	64.8 (60.8–69.1)
MEM/avibactam (4:1)	32^*b*^	77.5 (64.4–93.1)
**Experiment 2**		
MEM	32	118 (ND)
MEM/KSP-1007 (4:1)	0.03^*b*^	15.7 (ND)
MEM/taniborbactam (4:1)	0.12^*b*^	50.9 (34.5–168)
***A. baumannii*** **CDC-83 (NDM-1, OXA-23, 69)**		
MEM	128	ND (401^*d*^)
MEM/KSP-1007 (1:1)	2^*c*^	16.3 (10.7–22.0)
***A. baumannii*** **CDC-303 (OXA-23, 66)**		
MEM	16	ND (6,550^*d*^)
MEM/KSP-1007 (1:1)	1^*c*^	73.7 (53.5–105)
***P. aeruginosa*** **CDC-90 (KPC-5)**		
MEM	32	54.8 (35.8–83.9)
MEM/KSP-1007 (1:1)	0.5^*c*^	6.03 (3.28–10.0)
***P. aeruginosa*** **CDC-110 (VIM-2)**		
MEM	64	218 (170–346)
MEM/KSP-1007 (1:1)	16^*c*^	50.9 (36.5–65.3)

^*a*^ ND, not determined.

^*b*^ Fixed concentration at 4 μg/mL.

^*c*^ Fixed concentration at 8 μg/mL.

^*d*^ Values in parentheses were extrapolated from the probit analysis.

The 50% effective dose (ED_50_) of MEM under the KSP-1007 quarter dose combination against infection with KPC-producing *K. pneumoniae* was comparable to those of MEM in combination with vaborbactam, avibactam, or taniborbactam (ratio 4/1) and was much lower than that of MEM alone. The ED_50_ of MEM/KSP-1007 against infection with NDM-producing *E. coli* was lower than those of MEM alone, MEM/vaborbactam, MEM/avibactam, and MEM/taniborbactam at a dose ratio of 4:1. These results indicate that the *in vivo* activities of KSP-1007, vaborbactam, avibactam, and taniborbactam against KPC-2 and NDM-1 producers in a lethal infection model correlated with their *in vitro* activity.

The ED_50_s of MEM alone were 54.8 to >300 mg/kg against infection with CPAB and CPPA. KSP-1007 addition lowered MEM ED_50_ for *A. baumannii* by 25-fold for NDM- and OXA-producing and 89-fold for only OXA-producing, while for *P. aeruginosa,* it was lowered 9-fold for KPC-producing and 4-fold for VIM-producing.

KSP-1007 enhanced the *in vivo* activity of MEM against KPC, MBL, and/or OXA enzyme-producing GNB.

### *In vivo* efficacy against CPE in neutropenic murine complicated urinary tract infection models

**Fig 2:**
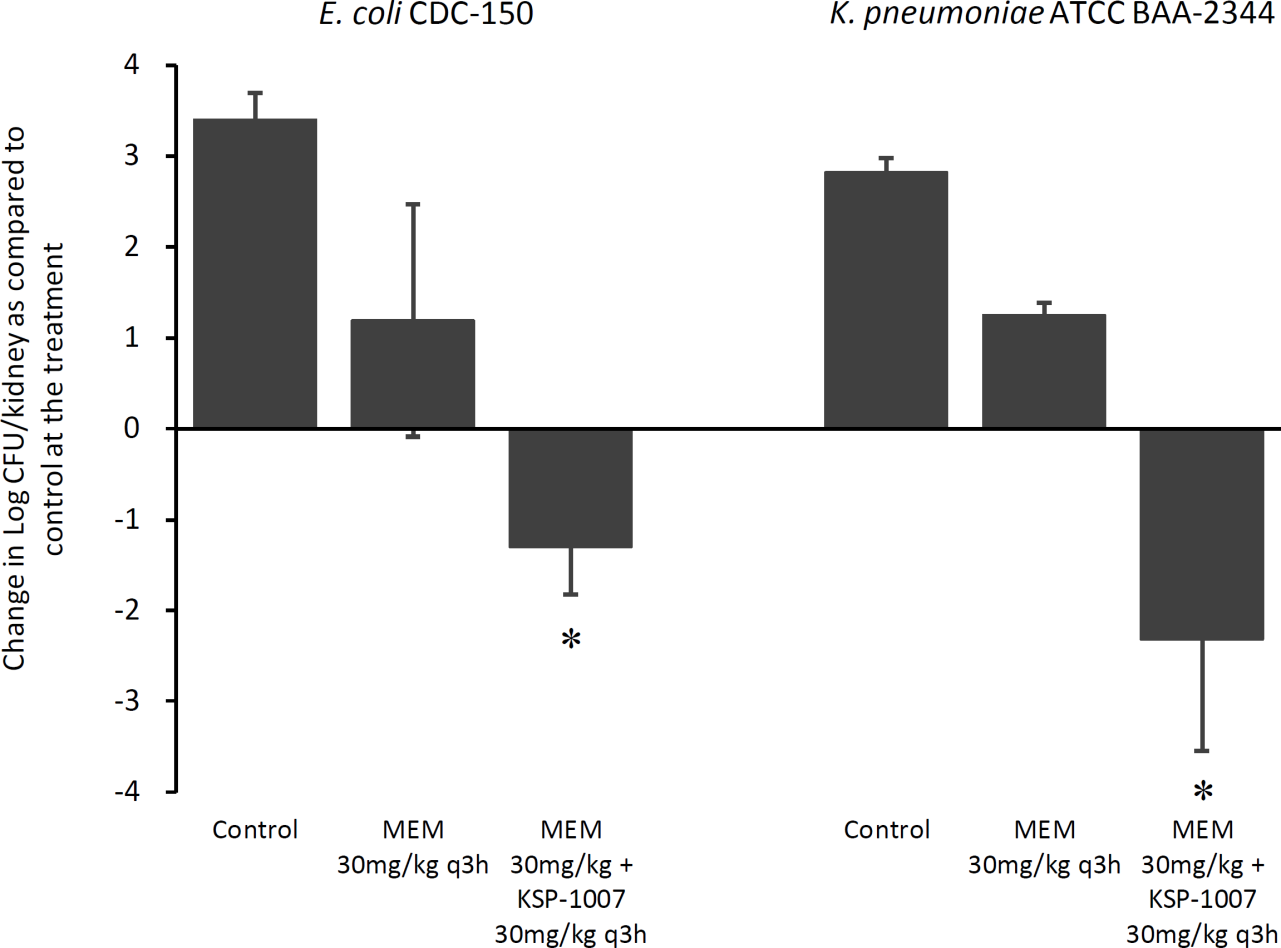
Activities of MEM in combination with KSP-1007 against *E. coli* CDC-150 (NDM-5 producer) and *K. pneumoniae* ATCC BAA-2344 (KPC-2 producer) in neutropenic murine complicated urinary tract infection models. The bacterial growth or reduction in log_10_ CFU/kidney plus and minus the standard deviation at 24 h relative to the start of treatment. The mean renal bacterial burdens at the start of treatment were 5.30 log_10_ CFU/kidney and 5.99 log_10_ CFU/kidney in the *E. coli* CDC-150 and *K. pneumoniae* ATCC BAA-2344 infection models, respectively. Treatments started approximately 4 h after infection and continued for 24 h subcutaneously (*n* = 4). ^*^Significantly different from MEM 30 mg/kg q3h (*P* < 0.05 by the Welch test).

For both the *E. coli* CDC-150 (NDM-5 producer and PBP3 mutant) and *K. pneumoniae* ATCC BAA-2344 (KPC-2 producer) infection models, the control groups, MEM 30 mg/kg every 3 h (q3h)-treated groups, and MEM 30 mg/kg plus KSP-1007 30 mg/kg q3h-treated groups resulted in about 3-log_10_ growth, 1-log_10_ growth, and more than a 1-log_10_ reduction in the kidney, respectively, relative to the level at the start of treatment (Fig. 2). Treatment with MEM/KSP-1007 resulted in more than a 2-log_10_ decrease compared to the use of MEM alone (*P* < 0.05). KSP-1007 improved the MEM efficacy in murine complicated urinary tract infection (cUTI) models using strains harboring serine- or metallo-type carbapenemase.

### *In vivo* efficacy against CPAB in a neutropenic murine thigh infection model

**Fig 3:**
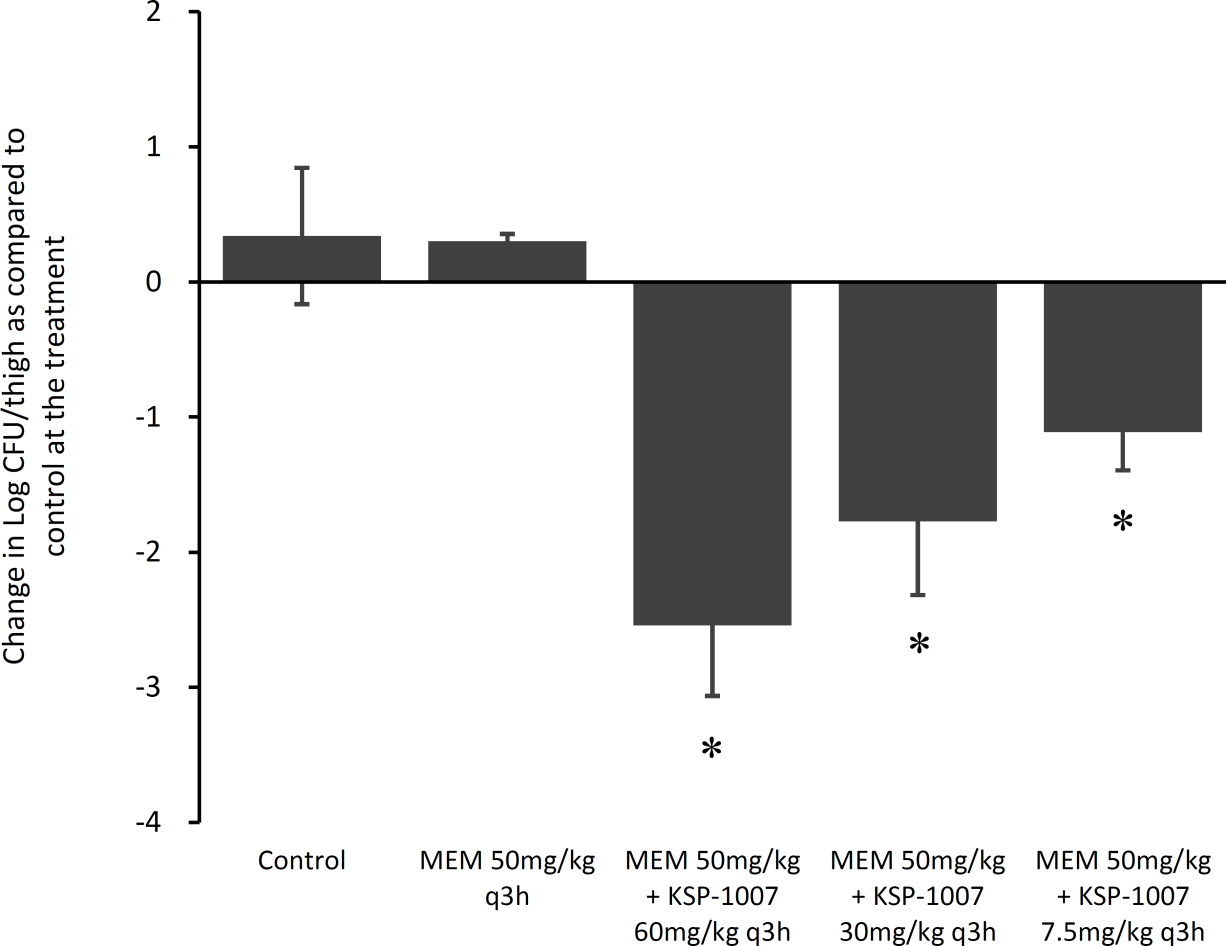
MEM activity at 50 mg/kg q3h alone and in combination with KSP-1007 against *A. baumannii* CDC-35 (OXA-66 and OXA-72 producer) in a neutropenic murine thigh infection model. The bacterial growth or reduction in log_10_ CFU/thigh plus or minus the standard deviation at 24 h relative to the start of treatment. The mean bacterial burden in the thighs at the start of treatment was 7.50 log_10_ CFU/thigh. Treatments began 2 h after infection and continued for 24 h subcutaneously (*n* = 4). ^*^Significantly different from MEM 50 mg/kg q3h (*P* < 0.005 by the Welch test).

Against the *A. baumannii* CDC-35 (OXA-66 and OXA-72 producer) infection model, the MEM 50 mg/kg q3h-treated group showed no significant reduction in bacterial growth in the thigh compared with the control group (Fig. 3). The combination treatment groups with KSP-1007 showed a dose-dependent reduction of bacterial growth in the thigh compared with MEM alone (*P* < 0.005) and control, yielding more than a 2-log_10_ reduction at ≥30 mg/kg q3h.

## DISCUSSION

β-Lactam antibiotics, due to their safety, efficacy, and broad activity spectrum, have long played a central role in the treatment of bacterial infections. Against β-lactamase-producing bacteria, a problem worldwide, one promising approach is to use BLI to restore β-lactam antibiotic efficacy. However, no approved β-lactam/BLI combination has shown efficacy against GNB-producing MBLs such as IMPs and NDMs, nor are these agents active against OXA enzyme (e.g., OXA-23) producers like *A. baumannii*. To address this unmet medical need, KSP-1007, a novel broad-spectrum BLI, is being developed for combination treatments with MEM for serious infections caused by multidrug-resistant GNB, including carbapenemase-producing organisms (20). Indeed, *in vitro* and *in vivo* data demonstrated that KSP-1007 improved MEM efficacy against CR-GNBs that produce both serine- and metallo-type carbapenemases.

The biochemical characterization studies demonstrated a broader inhibitory spectrum for KSP-1007 against carbapenemases than taniborbactam, which also inhibits serine- and metallo-type carbapenemases (21, 22). In addition to the *K*_*i* app_ against carbapenemases, KSP-1007 also potentiated the antimicrobial activity of MEM ≥8-fold against *Enterobacterales*, *A. baumannii*, and *P. aeruginosa* harboring each carbapenemase gene (KPC-4, 5, 6, and 11; OXA-71, 82, 181, and 232; SME-3; NMC-A; VIM-27; and IMP-4, 8, and 34) (see Tables S1 and S2 in the supplemental material), implying KSP-1007 inhibition of those carbapenemases as well.

The inhibitory activities of KSP-1007 against extended-spectrum β-lactamases (ESBLs) (CTX-Ms, SHVs, and TEM-10), which have limited MEM degradation capabilities, were lower than those of taniborbactam, whereas KSP-1007 demonstrated superior carbapenemase-inhibitory activities compared to taniborbactam. These findings suggest that MEM is a more suitable β-lactam partner for KSP-1007 than cephalosporins, which are degraded by ESBLs. The activities of MEM/KSP-1007 against MBL-producing *Enterobacterales* and *A. baumannii* were superior to those of cefepime/KSP-1007, supporting the selection of MEM as a partner β-lactam.

A phase 1 clinical trial of MEM (2 g every 8 h as a 3-h infusion) plus KSP-1007 was conducted (20). The pharmacokinetic/pharmacodynamic breakpoint MIC for MEM in this dosing regimen is considered 8 µg/mL (23, 24). At a fixed concentration of 8 µg/mL KSP-1007, the MEM MIC_90_s against CPE and *A. baumannii* as well as MEM MICs against some isolates of *P. aeruginosa* were reduced below the breakpoint MIC (≤8 µg/mL). The free concentration of 30 mg/kg of KSP-1007 observed to be effective in the urinary tract and thigh infection models exceeded 8 µg/mL (data not shown). For the *in vitro* microdilution MIC method, a fixed test concentration of KSP-1007 of 8 µg/mL would be appropriate, although, for future studies, large-scale susceptibility testing of GNB, PK/PD analysis of an animal infection model, and human pharmacokinetic parameters are required to confirm the validity of 8 µg/mL as a fixed test concentration.

The presence of PBP3 mutations and NDM carbapenemase are reported to decrease *Enterobacterales* susceptibility to the new drug, cefiderocol, and aztreonam/avibactam and cefepime/taniborbactam, which are in clinical development (16–18, 25, 26). Consistent with these reports, cefiderocol, aztreonam/avibactam, and cefepime/taniborbactam had relatively low activities against some isolates of *E. coli* with NDM and PBP3 mutations, with median MICs of 4, 2, and 16 µg/mL, respectively. For these isolates, the MICs of MEM with KSP-1007 at 4 µg/mL were ≤0.5 µg/mL. Furthermore, MEM/KSP-1007 showed good activity against *E. coli* CDC-150 with PBP3 mutations and NDM-5 in the cUTI model, implying that, in refractory cases, MEM/KSP-1007 may be an effective treatment alternative to monobactam- or cephalosporin-based therapy.

Few β-lactam/BLIs have efficacy against CRAB infections. A recent report indicates that sulbactam/durlobactam is not effective against MBL-producing *A. baumannii* (27). Cefiderocol is one therapeutic option for CRAB infections, but it had a higher mortality rate than the best available therapy in the CREDIBLE-CR study (12). This appears associated with both CRAB infections and reduced cefiderocol susceptibility (12). Cefiderocol resistance is associated with reduced expression of pirA siderophore receptor, amino acid substitution, and insertion of four amino acids in PBP3, PER-like β-lactamases, or NDM-like β-lactamases (28–30). As new agents are used clinically, resistance may develop, necessitating additional treatment options to address drug-resistant bacteria. In our experimental results, OXA- and NDM-producing *A. baumannii* CDC-83 was resistant to cefiderocol with a MIC of 16 µg/mL, while MEM/KSP-1007 at 8 µg/mL was effective against this strain with a MIC of 2 µg/mL. We believe that MEM/KSP-1007 is a viable carbapenem-based treatment candidate for OXA- and NDM-producing CRAB infections.

KSP-1007 only exhibited a slight influence on MEM efficacy against CRPA. The insufficient efficacy of MEM/KSP-1007 against *P. aeruginosa* is thought to result from the efflux of MEM by efflux pumps and low MEM outer membrane permeability caused by porin dysfunction and different resistance mechanisms to those related to β-lactamase production (31, 32). However, as we plan to develop MEM treatments using a higher dose (6 g/day), we expect clinical efficacy enhancements against *P. aeruginosa* infections over the usual dose (3 g/day).

The bicyclic boronate xeruborbactam is also a broad-spectrum inhibitor of serine and metallo-β-lactamases, with a high protein binding rate of >90% at a dose up to 1,000 mg (10). In comparison, the human plasma protein binding rate of KSP-1007 is about 75% (33). As the human protein binding of MEM is relatively low at 2% (34), it is a suitable combination drug to investigate the effects of human protein binding on BLI efficacy. Although the *in vitro* activities of KSP-1007 in combination with MEM against CPE and CPAB were similar or inferior to those of xeruborbactam, KSP-1007 activity, unlike xeruborbactam, was unaffected by the addition of 4% HSA (see Table S3 in the supplemental material) or 50% of human plasma (35). Thus, the physicochemical properties may differ between the two inhibitors.

In conclusion, *in vitro* and *in vivo* efficacy studies demonstrated that KSP-1007 broadly inhibited serine- and metallo-β-lactamases and increased MEM efficacy against CR-GNB that produce them. The combination of MEM and KSP-1007 was also effective against CR-GNB with low susceptibility to currently approved and investigational drugs in development and is expected to be a new and effective carbapenem-based treatment option for infections caused by CR-GNB.

## MATERIALS AND METHODS

### Organisms

Three hundred and five isolates were obtained from CDC and FDA Antibiotic Resistance Isolate Bank via the National Institute of Infectious Diseases, Japan. The collection included 154 isolates of *Enterobacterales* (61 producing only serine-carbapenemases and 47 producing MBLs), 55 isolates of *A. baumannii* (50 producing only serine-carbapenemases and 4 producing MBLs), and 96 isolates of *P. aeruginosa* (7 producing only serine-carbapenemases and 26 producing MBLs). From the National Collection of Type Cultures and the American Type Culture Collection (ATCC), 29 KPC, OXA, IMP, NDM, and VIM-producing strains were obtained, and 24 clinical isolates from Japan were also used (19 serine-carbapenemase-producing and 27 MBL-producing) (see Tx in the supplemental material). ATCC strains, NBRC strains from NITE Biological Resource Center, Gifu type collection strains, and Japanese clinical isolates were used as reference bacterial strains (see Tables S4 and S5 in the supplemental material).

### Antibacterial agents and BLIs

KSP-1007 and MEM hydrate were synthesized at Sumitomo Pharma Co., Ltd. Xeruborbactam was synthesized at Kitasato University. The other agents and inhibitors were obtained from commercial sources.

### β-Lactamase preparation

The coding sequences for β-lactamases, excluding the signal sequences, were cloned into a pDEST17 vector to create an expression construct with N-terminal 6×His tag. These plasmids were transformed into the *E. coli* BL21-AI strain. The transformant was cultured at 37°C in Luria–Bertani broth containing 100 µg/mL ampicillin. The culture was centrifuged, and the pellet was resuspended in 1/50 M phosphate buffer at pH 7 (PB). The bacterial suspension was lysed with lysozyme (100 µg/mL) and sonicated, with the supernatant collected by centrifugation at 8,000 × *g*. The bacterial lysate with 10 mM imidazole was loaded into a gravity flow column containing Ni Sepharose High Performance (GE Healthcare) pre-equilibrated with 10 mM imidazole in PB. After washing the column with PB containing 10 to 50 mM imidazole, the His-tagged β-lactamase was eluted with 100 to 500 mM imidazole in PB and dialyzed against PB using a Float-A-Lyzer G2 system (Funakoshi Ltd., Tokyo, Japan). An equal volume of glycerol was added to the β-lactamase solution and stored at −20°C until use.

### Determination of BLI *K_i_*_app_s against various β-lactamases

Serine-β-lactamases were mixed with inhibitors in PB with or without 0.01% bovine serum albumin, then incubated for 30 min at 37°C. For MBLs, 100 µM ZnSO_4_ was added to PB. Prewarmed nitrocefin or MEM at 25 to 100 µM was added (final inhibitor concentration, 0.3 nM to 100 µM), and the absorbance (490 or 297 nm) due to substrate degradation was measured every 30 to 60 seconds for 30 min at 37°C using the microplate reader. After the calculation of the initial reaction rate, *K_i_*_app_ values were determined by Waley’s method (36).

### MIC and MBC testing

MICs were determined by the broth microdilution method and agar dilution method (for anaerobic bacteria only) according to CLSI (37, 38). When investigating the effect of HSA on the MICs, CAMHB with 4% HSA (FUJIFILM Wako Pure Chemical Corporation) was used. After MIC determination, all medium was removed from each well showing no growth in the MIC testing plates, plated on Mueller Hinton agar (MHA), and incubated at 35°C for 18 h. The MBC was defined as the lowest drug concentration at which ≥99.9% of the original inoculum was killed.

### Animals

All animal experiments were approved by the Institutional Animal Care and Use Committee of Ōmura Satoshi Memorial Institute, Kitasato University, and were performed in accordance with the regulations for animal experiments of the institute. Three- or four-week-old male and four-week-old female Slc:ICR mice were purchased from Japan SLC, Inc. (Shizuoka, Japan) and were used at 4 and 5 weeks of age.

### Murine systemic infection models

The organisms were grown overnight at 35°C–36°C on MHA. Next, cells were collected and suspended in saline to the desired concentrations. To prepare each bacterial suspension for infection, they were diluted 2-fold with 16% gastric mucin suspension. Male mice were then inoculated intraperitoneally with 0.2 mL of each bacterial suspension. Two hours after inoculation, mice were injected subcutaneously with a single dose of antibiotics and BLIs. At each administration, 100 mg/kg of cilastatin, a dehydropeptidase-I inhibitor, was co-administered to prevent MEM degradation by this rodent enzyme (39, 40). The ED_50_ and 95% confidence intervals were calculated by probit analysis of survivor numbers for each group on day 7 and each MEM dosage.

### Murine complicated urinary tract infection models

A murine model of complicated urinary tract infection using the direct inoculation method was established as previously described (41). The organisms were grown on MHA as described above. Next, cells were suspended in Luria–Bertani broth and incubated for 18.5 h at 35°C. The bacterial culture was diluted in sterile saline to the desired concentrations. To induce neutropenia, female mice were intraperitoneally administered 150 and 100 mg/kg cyclophosphamide on 4 and 1 day before infection, respectively. Under isoflurane anesthesia, neutropenic mice were inoculated with 0.05 mL of bacterial suspension into the left renal pelvis (2 × 10^4^ to 3 × 10^4^ CFU/mouse) after shaving and incision of the area over the left kidney approximately 4 h before therapy. The incision was closed with a 6–0 silk suture and an 11-mm needle. Infected mice were subcutaneously treated with MEM at 30 mg/kg with and without KSP-1007 at 30 mg/kg q3h for 24 h. The control group was subcutaneously administered sterile saline q3h for 24 h. The renal bacterial burden was determined approximately 4 and 28 h after infection. Mice were sacrificed by bleeding from the heart under isoflurane anesthesia. The kidney was excised and homogenized in 0.7 mL of sterile saline with a Multi-Beads shocker (Yasui Kikai Co.). About 10-fold serial dilutions of kidney homogenates were prepared with sterile saline with 0.1 mL of each dilution plated onto MHA. The plates were incubated at 35°C for 1 day and colonies then enumerated.

### Murine thigh infection models

*A. baumannii* CDC-35 was grown on MHA as described above. Next, cells were suspended in sterile saline and adjusted to 0.5 McFarland. An overnight culture was prepared by inoculating 4 mL CAMHB with 40 µL of the bacterial suspension, at 36°C with agitation at 40 rpm. Then, the overnight culture was diluted with fresh CAMHB, adjusted to 1.5 McFarland, and incubated for 2 h at 36°C with agitation at 40 rpm. The bacterial culture was diluted in sterile saline to achieve a concentration of approximately 1 × 10^7^ CFU/mL. As previously described (42), cyclophosphamide-induced neutropenic male mice were inoculated with 0.1 mL of the bacterial suspensions into the left gastrocnemius muscles (approximately 1 × 10^6^ CFU/mouse) at 2 h before therapy. Infected mice were subcutaneously treated with MEM at 50 mg/kg with and without KSP-1007 at 7.5–60 mg/kg q3h for 24 h. At each administration, 100 mg/kg of cilastatin was co-administered. The control group was subcutaneously administered sterile saline every 3 h for 24 h. The bacterial burden in the thighs was determined 2 and 26 h after infection in the same method as the renal bacterial counts described above.

### Statistical analysis

Change in log CFU per tissue at 24 h was compared using the Welch test with a *P*-value <0.05 considered significant. Statistical analyses were performed using the SAS program in the Stat Preclinica (Takumi Information Technology Inc., Tokyo, Japan).

## SUPPLEMENTAL MATERIAL

Tables S1 to S5 aac.01602-23-s0001.pdf MEM/KSP-1007 MICs against Gram-negative bacteria, the effects of 4% HSA on *in vitro* activity, and the MICs of KSP-1007 alone.

ASM does not own the copyrights to Supplemental Material that may be linked to, or accessed through, an article. The authors have granted ASM a non-exclusive, world-wide license to publish the Supplemental Material files. Please contact the corresponding author directly for reuse.
