# Supplementary material for: In vitro and in vivo activities of KSP-1007, a broad-spectrum inhibitor of serine- and metallo-β-lactamases, in combination with meropenem against carbapenem-resistant Gram-negative bacteria
Source: Antimicrob Agents Chemother. 2024 May 6;68(6):e01602-23. doi: 10.1128/aac.01602-23 (PMC11620503; doi:10.1128/aac.01602-23)
Supplement: Tables S1 to S5 — MEM/KSP-1007 MICs against Gram-negative bacteria, the effects of 4% HSA on in vitro activity, and the MICs of KSP-1007 alone. [file aac.01602-23-s0001.pdf]

Table S1. The MIC values of meropenem alone and meropenem/KSP-1007 against Gram-negative bacteria

Organism	AR Bank #	Harbored carbapenemase genes		MIC of meropenem (μg/mL)			
		Serine-type	Metallo-type	Meropenem	Meropenem/ KSP-1007 4 μg/mL	Meropenem/ KSP-1007 8 μg/mL	Meropenem/ KSP-1007 16 μg/mL
Enterobacter cloacae	32	KPC-3	-	0.12	≤0.06	≤0.06	≤0.06
Klebsiella pneumoniae	34	-	IMP-4	0.25	≤0.06	≤0.06	≤0.06
Enterobacter cloacae	38	-	NDM-1	>32	>32	>32	>32
Klebsiella pneumoniae	39	OXA-181	-	1	≤0.06	≤0.06	≤0.06
Klebsiella pneumoniae	40	-	VIM-27	>32	16	8	1
Klebsiella pneumoniae	41	-	NDM-1	8	≤0.06	≤0.06	≤0.06
Klebsiella pneumoniae	43	-	-	0.5	0.5	0.25	0.12
Klebsiella pneumoniae	44	-	-	2	0.25	0.25	0.12
Klebsiella pneumoniae	46	-	VIM-27	32	8	2	0.5
Klebsiella pneumoniae	47	-	-	4	2	1	0.5
Escherichia coli	48	-	NDM-1	16	≤0.06	≤0.06	≤0.06
Klebsiella pneumoniae	49	-	NDM-1	32	0.25	≤0.06	≤0.06
Enterobacter cloacae	50	KPC-4	-	0.5	≤0.06	≤0.06	≤0.06
Klebsiella ozaenae	51	OXA-181	-	1	≤0.06	≤0.06	≤0.06
Enterobacter cloacae	53	KPC-3	-	2	≤0.06	≤0.06	≤0.06
Escherichia coli	55	-	NDM-1	2	≤0.06	≤0.06	≤0.06
Morganella morganii	57	-	NDM-1	2	≤0.06	≤0.06	≤0.06
Escherichia coli	58	-	-	0.25	≤0.06	≤0.06	≤0.06
Proteus mirabilis	59	-	-	0.12	0.12	0.12	0.12
Enterobacter cloacae	60	-	-	≤0.06	≤0.06	≤0.06	≤0.06
Escherichia coli	61	KPC-3	-	1	≤0.06	≤0.06	≤0.06
Enterobacter aerogenes	62	-	-	0.12	≤0.06	≤0.06	≤0.06
Enterobacter cloacae	65	-	-	0.25	≤0.06	≤0.06	≤0.06
Klebsiella pneumoniae	66	OXA-232	-	8	1	0.25	0.25
Escherichia coli	67	-	-	≤0.06	≤0.06	≤0.06	≤0.06
Klebsiella pneumoniae	68	OXA-232	NDM-1	>32	32	1	0.25
Escherichia coli	69	-	NDM-1	1	≤0.06	≤0.06	≤0.06
Klebsiella oxytoca	71	-	-	8	8	2	0.25
Enterobacter cloacae	72	-	-	0.12	≤0.06	≤0.06	≤0.06
Enterobacter cloacae	73	-	-	≤0.06	≤0.06	≤0.06	≤0.06
Enterobacter aerogenes	74	OXA-48	-	0.5	≤0.06	≤0.06	≤0.06

Organism	AR Bank #	Harbored carbapenemase genes		MIC of meropenem (μg/mL)			
		Serine-type	Metallo-type	Meropenem	Meropenem/ KSP-1007 4 μg/mL	Meropenem/ KSP-1007 8 μg/mL	Meropenem/ KSP-1007 16 μg/mL
Klebsiella pneumoniae	75	OXA-232	-	8	1	≤0.06	≤0.06
Klebsiella pneumoniae	76	-	VIM-1	2	≤0.06	≤0.06	≤0.06
Escherichia coli	77	-	-	≤0.06	≤0.06	≤0.06	≤0.06
Klebsiella pneumoniae	79	-	-	4	4	2	0.25
Klebsiella pneumoniae	80	-	IMP-4	4	0.25	≤0.06	≤0.06
Escherichia coli	81	-	-	≤0.06	≤0.06	≤0.06	≤0.06
Providencia rettgeri	82	-	NDM-1	8	≤0.06	≤0.06	≤0.06
Escherichia coli	84	-	-	≤0.06	≤0.06	≤0.06	≤0.06
Escherichia coli	85	-	-	0.25	≤0.06	≤0.06	≤0.06
Escherichia coli	86	-	-	0.12	≤0.06	≤0.06	≤0.06
Klebsiella pneumoniae	87	-	-	≤0.06	≤0.06	≤0.06	≤0.06
Escherichia coli	89	-	-	≤0.06	≤0.06	≤0.06	≤0.06
Serratia marcescens	91	SME-3	-	16	≤0.06	≤0.06	≤0.06
Enterobacter cloacae	93	KPC-6	-	1	≤0.06	≤0.06	≤0.06
Klebsiella ozaenae	96	KPC-3	-	32	≤0.06	≤0.06	≤0.06
Klebsiella pneumoniae	97	KPC-3	-	32	≤0.06	≤0.06	≤0.06
Klebsiella pneumoniae	98	KPC-2	-	8	≤0.06	≤0.06	≤0.06
Serratia marcescens	99	SME-3	-	16	≤0.06	≤0.06	≤0.06
Escherichia coli	104	KPC-4	-	0.5	≤0.06	≤0.06	≤0.06
Klebsiella pneumoniae	106	-	NDM-1	>32	>32	2	1
Klebsiella pneumoniae	107	-	-	16	4	0.5	0.12
Klebsiella pneumoniae	109	-	-	8	4	1	0.25
Klebsiella pneumoniae	112	KPC-3	-	4	≤0.06	≤0.06	≤0.06
Klebsiella pneumoniae	113	KPC-3	-	>32	16	0.12	0.12
Escherichia coli	114	KPC-3	-	2	≤0.06	≤0.06	≤0.06
Klebsiella pneumoniae	115	KPC-3	-	8	≤0.06	≤0.06	≤0.06
Citrobacter freundii	116	KPC-2	-	16	≤0.06	≤0.06	≤0.06
Klebsiella pneumoniae	117	KPC-3	-	16	≤0.06	≤0.06	≤0.06
Escherichia coli	118	-	NDM-1	32	≤0.06	≤0.06	≤0.06
Escherichia coli	119	-	NDM-1	32	≤0.06	≤0.06	≤0.06
Klebsiella pneumoniae	120	KPC-2	-	16	≤0.06	≤0.06	≤0.06
Serratia marcescens	121	SME-3	-	16	≤0.06	≤0.06	≤0.06

Organism	AR Bank #	Harbored carbapenemase genes		MIC of meropenem (µg/mL)			
		Serine-type	Metallo-type	Meropenem	Meropenem/ KSP-1007 4 µg/mL	Meropenem/ KSP-1007 8 µg/mL	Meropenem/ KSP-1007 16 µg/mL
Serratia marcescens	122	SME-3	-	32	≤0.06	≤0.06	≤0.06
Serratia marcescens	123	SME-3	-	2	≤0.06	≤0.06	≤0.06
Serratia marcescens	124	SME-3	-	32	≤0.06	≤0.06	≤0.06
Klebsiella pneumoniae	125	KPC-3	-	16	≤0.06	≤0.06	≤0.06
Klebsiella pneumoniae	126	KPC-2	-	4	≤0.06	≤0.06	≤0.06
Salmonella Senftenberg	127	-	NDM-1	16	≤0.06	≤0.06	≤0.06
Escherichia coli	128	-	NDM-1	32	≤0.06	≤0.06	≤0.06
Klebsiella pneumoniae	129	KPC-3	-	4	≤0.06	≤0.06	≤0.06
Serratia marcescens	130	SME-3	-	8	≤0.06	≤0.06	≤0.06
Serratia marcescens	131	SME-3	-	0.5	≤0.06	≤0.06	≤0.06
Enterobacter cloacae group	132	NMC-A	-	2	≤0.06	≤0.06	≤0.06
Morganella morganii	133	KPC-2	-	1	≤0.06	≤0.06	≤0.06
Raoultella ornithinolytica	134	KPC-3	-	0.25	≤0.06	≤0.06	≤0.06
Klebsiella pneumoniae	135	-	VIM-1	0.5	≤0.06	≤0.06	≤0.06
Enterobacter cloacae	136	KPC-3	-	8	≤0.06	≤0.06	≤0.06
Escherichia coli	137	-	NDM-6	32	0.12	0.12	0.12
Klebsiella pneumoniae	138	-	NDM-7	>32	>32	32	1
Klebsiella pneumoniae	139	-	NDM-1	0.12	≤0.06	≤0.06	≤0.06
Klebsiella pneumoniae	140	OXA-181	-	1	≤0.06	≤0.06	≤0.06
Klebsiella pneumoniae	141	OXA-181	-	2	≤0.06	≤0.06	≤0.06
Klebsiella pneumoniae	142	OXA-181	-	1	≤0.06	≤0.06	0.12
Klebsiella pneumoniae	143	-	NDM-1	>32	32	8	1
Kluyvera ascorbata	144	KPC-3	-	2	≤0.06	≤0.06	≤0.06
Klebsiella pneumoniae	145	-	NDM-1	8	≤0.06	≤0.06	≤0.06
Klebsiella pneumoniae	146	-	NDM-1	8	≤0.06	≤0.06	≤0.06
Klebsiella oxytoca	147	KPC-3	-	0.25	≤0.06	≤0.06	≤0.06
Klebsiella pneumoniae	148	-	NDM-1	8	≤0.06	≤0.06	≤0.06
Escherichia coli	149	-	NDM-7	8	0.5	0.5	0.5
Escherichia coli	150	-	NDM-5	>32	≤0.06	≤0.06	≤0.06
Escherichia coli	151	-	NDM-5	8	≤0.06	≤0.06	≤0.06
Klebsiella pneumoniae	152	-	NDM-1	8	≤0.06	≤0.06	≤0.06
Klebsiella pneumoniae	153	OXA-232	NDM-1	>32	16	1	0.5

Organism	AR Bank #	Harbored carbapenemase genes		MIC of meropenem (μg/mL)			
		Serine-type	Metallo-type	Meropenem	Meropenem/ KSP-1007 4 μg/mL	Meropenem/ KSP-1007 8 μg/mL	Meropenem/ KSP-1007 16 μg/mL
Enterobacter cloacae	154	-	VIM-1	4	≤0.06	≤0.06	≤0.06
Proteus mirabilis	155	KPC-6	-	1	≤0.06	≤0.06	≤0.06
Proteus mirabilis	156	KPC-2	-	0.12	≤0.06	≤0.06	≤0.06
Citrobacter freundii	157	-	NDM-1	32	0.12	0.12	0.12
Klebsiella pneumoniae	158	-	NDM-1	8	≤0.06	≤0.06	≤0.06
Proteus mirabilis	159	-	NDM-1	2	≤0.06	≤0.06	≤0.06
Klebsiella pneumoniae	160	OXA-48	-	8	≤0.06	≤0.06	≤0.06
Enterobacter aerogenes	161	-	IMP-4	1	≤0.06	≤0.06	≤0.06
Escherichia coli	162	-	NDM-7	32	≤0.06	≤0.06	≤0.06
Enterobacter cloacae	163	KPC-2	-	4	≤0.06	≤0.06	≤0.06
Enterobacter cloacae complex	164	NMC-A	-	32	≤0.06	≤0.06	≤0.06
Klebsiella pneumoniae	361	KPC-2	-	8	≤0.06	≤0.06	≤0.06
Klebsiella pneumoniae	362	KPC-2	-	8	≤0.06	≤0.06	≤0.06
Klebsiella pneumoniae	363	KPC-2	-	4	≤0.06	≤0.06	≤0.06
Klebsiella pneumoniae	364	-	-	1	≤0.06	≤0.06	≤0.06
Enterobacter cloacae	365	KPC-2	-	2	≤0.06	≤0.06	≤0.06
Enterobacter cloacae	366	KPC-2	-	1	≤0.06	≤0.06	≤0.06
Enterobacter cloacae	367	-	-	0.5	0.12	≤0.06	≤0.06
Escherichia coli	368	-	-	≤0.06	≤0.06	≤0.06	≤0.06
Escherichia coli	369	-	-	≤0.06	≤0.06	≤0.06	≤0.06
Escherichia coli	370	-	-	≤0.06	≤0.06	≤0.06	≤0.06
Escherichia coli	371	-	-	≤0.06	≤0.06	≤0.06	≤0.06
Escherichia coli	372	-	-	≤0.06	≤0.06	≤0.06	≤0.06
Escherichia coli	373	-	-	≤0.06	≤0.06	≤0.06	≤0.06
Escherichia coli	374	-	-	≤0.06	≤0.06	≤0.06	≤0.06
Klebsiella oxytoca	375	-	-	≤0.06	≤0.06	≤0.06	≤0.06
Klebsiella pneumoniae	376	-	-	0.12	≤0.06	≤0.06	≤0.06
Proteus mirabilis	377	KPC-3	-	1	≤0.06	≤0.06	≤0.06
Escherichia coli	378	-	-	≤0.06	≤0.06	≤0.06	≤0.06
Proteus mirabilis	379	-	-	≤0.06	≤0.06	≤0.06	≤0.06
Klebsiella oxytoca	380	-	-	2	0.5	0.25	0.25
Enterobacter aerogenes	431	-	-	≤0.06	≤0.06	≤0.06	≤0.06

Organism	AR Bank #	Harbored carbapenemase genes		MIC of meropenem (µg/mL)			
		Serine-type	Metallo-type	Meropenem	Meropenem/ KSP-1007 4 µg/mL	Meropenem/ KSP-1007 8 µg/mL	Meropenem/ KSP-1007 16 µg/mL
Enterobacter cloacae	432	-	-	0.12	≤0.06	≤0.06	≤0.06
Escherichia coli	433	-	-	≤0.06	≤0.06	≤0.06	≤0.06
Escherichia coli	434	-	-	≤0.06	≤0.06	≤0.06	≤0.06
Escherichia coli	435	-	NDM-1	32	≤0.06	≤0.06	≤0.06
Escherichia coli	436	-	-	≤0.06	≤0.06	≤0.06	≤0.06
Escherichia coli	437	-	-	≤0.06	≤0.06	≤0.06	≤0.06
Klebsiella pneumoniae	438	KPC-3	-	>32	32	1	0.12
Enterobacter cloacae	448	-	NDM-1	16	0.12	≤0.06	≤0.06
Escherichia coli	450	-	-	16	16	8	1
Escherichia coli	451	KPC-3	-	0.25	≤0.06	≤0.06	≤0.06
Escherichia coli	452	-	NDM-5	2	≤0.06	≤0.06	≤0.06
Klebsiella pneumoniae	453	KPC-3	-	>32	>32	16	0.5
Escherichia coli	861	-	NDM	32	0.12	0.12	≤0.06
Enterobacter cloacae	501	-	VIM-1	0.5	≤0.06	≤0.06	≤0.06
Enterobacter cloacae	502	-	IMP-8	4	2	1	0.25
Klebsiella pneumoniae	504	OXA-48	-	4	≤0.06	≤0.06	≤0.06
Klebsiella pneumoniae	505	-	NDM-1	16	≤0.06	≤0.06	≤0.06
Klebsiella pneumoniae	506	-	NDM-1	16	≤0.06	≤0.06	≤0.06
Klebsiella pneumoniae	507	OXA-232	NDM-1	>32	>32	>32	2
Serratia marcescens	517	KPC-3	-	8	≤0.06	≤0.06	≤0.06
Morganella morganii	519	-	-	2	2	1	1
Serratia marcescens	520	-	-	≤0.06	≤0.06	≤0.06	≤0.06
Serratia marcescens	521	-	-	≤0.06	≤0.06	≤0.06	≤0.06
Klebsiella pneumoniae	522	KPC-2	-	8	≤0.06	≤0.06	≤0.06
Klebsiella pneumoniae	523	KPC-2	-	16	0.12	≤0.06	≤0.06
Klebsiella pneumoniae	524	KPC-3	-	16	≤0.06	≤0.06	≤0.06
Klebsiella pneumoniae	525	KPC-11	-	8	≤0.06	≤0.06	≤0.06
Acinetobacter baumannii	33	OXA-94	NDM-1	128	4	2	1
Acinetobacter baumannii	35	OXA-66, OXA-72	-	128	8	4	1
Acinetobacter baumannii	36	OXA-24, OXA-65	-	64	4	2	1
Acinetobacter baumannii	37	OXA-94	NDM-1	128	4	2	1
Acinetobacter baumannii	45	OXA-23, OXA-69	-	16	2	1	0.5

Organism	AR Bank #	Harbored carbapenemase genes		MIC of meropenem (µg/mL)			
		Serine-type	Metallo-type	Meropenem	Meropenem/ KSP-1007 4 µg/mL	Meropenem/ KSP-1007 8 µg/mL	Meropenem/ KSP-1007 16 µg/mL
Acinetobacter baumannii	52	OXA-58, OXA-100	-	4	2	1	1
Acinetobacter baumannii	56	OXA-23, OXA-66	-	16	4	2	1
Acinetobacter baumannii	63	OXA-23, OXA-24, OXA-65	-	64	4	2	1
Acinetobacter baumannii	70	OXA-58, OXA-100	-	8	2	1	≤0.25
Acinetobacter baumannii	78	OXA-71	-	32	16	8	4
Acinetobacter baumannii	83	OXA-23, OXA-69	NDM-1	128	16	2	1
Acinetobacter baumannii	88	OXA-64	NDM-1	>128	4	2	0.5
Acinetobacter baumannii	101	OXA-24, OXA-65	-	128	2	1	1
Acinetobacter baumannii	102	OXA-66	-	2	1	1	1
Acinetobacter baumannii	273	OXA-23, OXA-66	-	32	4	4	2
Acinetobacter baumannii	274	OXA-66, OXA-72	-	>128	64	8	4
Acinetobacter baumannii	275	OXA-23, OXA-66	-	32	4	2	2
Acinetobacter baumannii	276	-	-	4	4	4	4
Acinetobacter baumannii	277	OXA-24, OXA-65	-	128	8	2	2
Acinetobacter baumannii	278	OXA-23, OXA-66	-	32	4	2	2
Acinetobacter baumannii	279	OXA-23, OXA-66	-	32	4	2	2
Acinetobacter baumannii	280	OXA-66	-	2	2	2	2
Acinetobacter baumannii	281	OXA-82	-	8	8	2	1
Acinetobacter baumannii	282	OXA-23, OXA-66	-	32	16	4	2
Acinetobacter baumannii	283	OXA-23, OXA-66	-	32	4	2	2
Acinetobacter baumannii	284	OXA-24, OXA-65	-	128	8	4	2
Acinetobacter baumannii	285	OXA-24, OXA-65	-	128	16	2	1
Acinetobacter baumannii	286	OXA-24, OXA-66	-	128	16	4	2
Acinetobacter baumannii	287	OXA-66, OXA-72	-	>128	128	32	2
Acinetobacter baumannii	288	OXA-23, OXA-66	-	16	4	4	2
Acinetobacter baumannii	289	OXA-66, OXA-72	-	128	32	8	4
Acinetobacter baumannii	290	OXA-23, OXA-66	-	32	8	4	4
Acinetobacter baumannii	291	OXA-23, OXA-66	-	32	2	4	1
Acinetobacter baumannii	292	OXA-66, OXA-72	-	>128	128	16	1
Acinetobacter baumannii	293	OXA-66, OXA-72	-	>128	128	16	1
Acinetobacter baumannii	294	OXA-23, OXA-65	-	32	8	4	1

Organism	AR Bank #	Harbored carbapenemase genes		MIC of meropenem (µg/mL)			
		Serine-type	Metallo-type	Meropenem	Meropenem/ KSP-1007 4 µg/mL	Meropenem/ KSP-1007 8 µg/mL	Meropenem/ KSP-1007 16 µg/mL
Acinetobacter baumannii	295	OXA-23, OXA-66	-	32	8	4	2
Acinetobacter baumannii	296	OXA-23, OXA-223	-	64	16	4	2
Acinetobacter baumannii	297	OXA-23, OXA-66	-	16	8	4	2
Acinetobacter baumannii	298	OXA-66, OXA-237	-	16	8	4	2
Acinetobacter baumannii	299	OXA-23, OXA-203	-	32	8	1	0.5
Acinetobacter baumannii	300	OXA-66	-	2	2	2	2
Acinetobacter baumannii	301	OXA-66, OXA-72	-	>128	64	8	2
Acinetobacter baumannii	302	OXA-23, OXA-82	-	32	16	4	2
Acinetobacter baumannii	303	OXA-23, OXA-66	-	16	2	1	0.5
Acinetobacter baumannii	304	OXA-66, OXA-72	-	64	1	2	1
Acinetobacter baumannii	305	OXA-24, OXA-65	-	128	4	4	1
Acinetobacter baumannii	306	OXA-24, OXA-65	-	128	8	4	2
Acinetobacter baumannii	307	OXA-66, OXA-237	-	8	2	2	1
Acinetobacter baumannii	308	OXA-71	-	16	4	2	1
Acinetobacter baumannii	309	OXA-23, OXA-82	-	32	8	4	2
Acinetobacter baumannii	310	OXA-23, OXA-82	-	32	8	4	2
Acinetobacter baumannii	311	OXA-23, OXA-82	-	32	8	4	2
Acinetobacter baumannii	312	OXA-69	-	4	4	4	4
Acinetobacter baumannii	313	OXA-23, OXA-69	-	16	2	1	0.5
Pseudomonas aeruginosa	54	-	VIM-4	>128	>128	>128	>128
Pseudomonas aeruginosa	64	-	SPM-1	128	128	128	128
Pseudomonas aeruginosa	90	KPC-5	-	32	4	0.5	≤0.25
Pseudomonas aeruginosa	92	-	IMP-14	>128	>128	>128	>128
Pseudomonas aeruginosa	94	-	-	16	16	16	16
Pseudomonas aeruginosa	95	-	-	8	8	8	8
Pseudomonas aeruginosa	100	-	VIM-2	128	64	64	32
Pseudomonas aeruginosa	103	-	IMP-1	>128	>128	>128	>128
Pseudomonas aeruginosa	105	-	-	4	4	4	2
Pseudomonas aeruginosa	108	-	VIM-2	128	64	32	16
Pseudomonas aeruginosa	110	-	VIM-2	64	32	16	8
Pseudomonas aeruginosa	111	-	VIM-2	32	32	8	8
Pseudomonas aeruginosa	229	-	-	32	32	32	32

Organism	AR Bank #	Harbored carbapenemase genes		MIC of meropenem (μg/mL)			
		Serine-type	Metallo-type	Meropenem	Meropenem/ KSP-1007 4 μg/mL	Meropenem/ KSP-1007 8 μg/mL	Meropenem/ KSP-1007 16 μg/mL
Pseudomonas aeruginosa	230	-	VIM-2	64	32	16	16
Pseudomonas aeruginosa	231	KPC-5	-	128	32	16	16
Pseudomonas aeruginosa	232	-	-	4	2	2	2
Pseudomonas aeruginosa	233	-	-	16	8	8	8
Pseudomonas aeruginosa	234	-	-	2	1	1	1
Pseudomonas aeruginosa	235	-	-	16	16	16	16
Pseudomonas aeruginosa	236	-	-	2	1	2	2
Pseudomonas aeruginosa	237	-	-	2	1	1	1
Pseudomonas aeruginosa	238	-	-	≤0.25	≤0.25	≤0.25	≤0.25
Pseudomonas aeruginosa	239	GES-1	VIM-11	64	64	64	32
Pseudomonas aeruginosa	240	-	VIM-2	>128	128	128	64
Pseudomonas aeruginosa	241	-	IMP-1	>128	>128	128	128
Pseudomonas aeruginosa	242	-	VIM-2	4	2	1	≤0.25
Pseudomonas aeruginosa	243	-	VIM-2	8	2	1	1
Pseudomonas aeruginosa	244	-	-	16	16	16	16
Pseudomonas aeruginosa	245	-	VIM-2	8	4	4	2
Pseudomonas aeruginosa	246	-	NDM-1	>128	>128	>128	>128
Pseudomonas aeruginosa	247	-	-	4	2	2	2
Pseudomonas aeruginosa	248	-	VIM-2	4	4	2	0.5
Pseudomonas aeruginosa	249	-	VIM-2	>128	>128	>128	64
Pseudomonas aeruginosa	250	-	NDM-1	>128	>128	>128	>128
Pseudomonas aeruginosa	251	-	-	1	1	1	1
Pseudomonas aeruginosa	252	-	-	2	2	2	1
Pseudomonas aeruginosa	253	-	-	0.5	0.5	1	0.5
Pseudomonas aeruginosa	254	-	VIM-2	64	64	16	8
Pseudomonas aeruginosa	255	-	VIM-2	>128	128	64	32
Pseudomonas aeruginosa	256	-	-	2	2	4	2
Pseudomonas aeruginosa	257	-	-	4	4	4	4
Pseudomonas aeruginosa	258	-	-	2	2	1	1
Pseudomonas aeruginosa	259	-	-	0.5	≤0.25	≤0.25	0.5
Pseudomonas aeruginosa	260	-	-	4	4	8	4
Pseudomonas aeruginosa	261	-	-	0.5	0.5	0.5	≤0.25

Organism	AR Bank #	Harbored carbapenemase genes		MIC of meropenem (μg/mL)			
		Serine-type	Metallo-type	Meropenem	Meropenem/ KSP-1007 4 μg/mL	Meropenem/ KSP-1007 8 μg/mL	Meropenem/ KSP-1007 16 μg/mL
Pseudomonas aeruginosa	262	-	-	0.5	0.5	0.5	0.5
Pseudomonas aeruginosa	263	-	-	≤0.25	≤0.25	≤0.25	≤0.25
Pseudomonas aeruginosa	264	-	-	2	2	2	2
Pseudomonas aeruginosa	265	-	-	8	8	8	8
Pseudomonas aeruginosa	266	-	-	8	8	8	4
Pseudomonas aeruginosa	267	-	-	2	2	2	2
Pseudomonas aeruginosa	268	-	-	16	16	8	8
Pseudomonas aeruginosa	269	-	-	8	8	8	8
Pseudomonas aeruginosa	270	-	-	2	2	2	2
Pseudomonas aeruginosa	271	-	-	8	8	8	8
Pseudomonas aeruginosa	272	-	-	2	1	2	2
Pseudomonas aeruginosa	351	-	-	16	16	16	16
Pseudomonas aeruginosa	352	-	-	128	128	128	128
Pseudomonas aeruginosa	353	GES-1	-	16	16	8	4
Pseudomonas aeruginosa	354	-	-	4	4	2	2
Pseudomonas aeruginosa	355	-	-	8	8	8	2
Pseudomonas aeruginosa	356	KPC-2	-	32	8	2	≤0.25
Pseudomonas aeruginosa	357	-	-	4	4	4	4
Pseudomonas aeruginosa	358	-	-	8	8	8	4
Pseudomonas aeruginosa	359	-	-	8	8	8	4
Pseudomonas aeruginosa	360	-	-	16	8	8	8
Pseudomonas aeruginosa	439	-	IMP-18	64	32	64	32
Pseudomonas aeruginosa	440	-	-	8	8	4	4
Pseudomonas aeruginosa	441	KPC-2	-	32	16	4	≤0.25
Pseudomonas aeruginosa	443	-	-	16	16	16	16
Pseudomonas aeruginosa	444	-	VIM-2	>128	>128	>128	128
Pseudomonas aeruginosa	445	-	-	64	64	64	32
Pseudomonas aeruginosa	447	-	-	2	2	2	2
Pseudomonas aeruginosa	449	-	-	16	16	8	8
Pseudomonas aeruginosa	455	-	-	16	16	16	16
Pseudomonas aeruginosa	456	-	-	64	64	64	64
Pseudomonas aeruginosa	457	-	VIM-2	128	128	64	32

Organism	AR Bank #	Harbored carbapenemase genes		MIC of meropenem (μg/mL)			
		Serine-type	Metallo-type	Meropenem	Meropenem/ KSP-1007 4 μg/mL	Meropenem/ KSP-1007 8 μg/mL	Meropenem/ KSP-1007 16 μg/mL
Pseudomonas aeruginosa	458	-	-	1	0.5	0.5	≤0.25
Pseudomonas aeruginosa	459	-	-	16	16	16	16
Pseudomonas aeruginosa	862	-	-	8	16	8	8
Pseudomonas aeruginosa	863	-	VIM	128	128	64	32
Pseudomonas aeruginosa	864	-	-	64	64	64	64
Pseudomonas aeruginosa	508	-	-	16	16	16	16
Pseudomonas aeruginosa	509	-	VIM-2	>128	>128	>128	64
Pseudomonas aeruginosa	510	-	-	0.5	0.5	≤0.25	≤0.25
Pseudomonas aeruginosa	511	-	-	4	4	4	4
Pseudomonas aeruginosa	512	-	-	16	16	16	16
Pseudomonas aeruginosa	513	-	-	32	32	16	16
Pseudomonas aeruginosa	514	-	-	4	4	4	4
Pseudomonas aeruginosa	515	-	-	32	32	32	16
Pseudomonas aeruginosa	516	KPC-2	-	64	32	16	1
Pseudomonas aeruginosa	518	KPC-2	-	>128	64	32	16
Pseudomonas aeruginosa	526	-	-	8	4	4	2
Pseudomonas aeruginosa	527	-	-	16	16	16	16
Pseudomonas aeruginosa	528	-	-	8	8	8	4
Pseudomonas aeruginosa	529	-	-	8	8	8	4

Table S2. The MIC values of meropenem alone and meropenem/KSP-1007 against *Enterobacterales*

Organism	Origin	Harbored carbapenemase genes	MIC of meropenem (µg/mL)			
			Meropenem	Meropenem/ KSP-1007 4 µg/mL	Meropenem/ KSP-1007 8 µg/mL	Meropenem/ KSP-1007 16 µg/mL
Escherichia coli	ATCC BAA-2340	KPC-3	0.5	0.015	0.015	0.015
Escherichia coli	ATCC BAA-2469	NDM-1	2	0.015	0.015	0.015
Escherichia coli	ATCC BAA-2523	OXA-48	0.06	0.015	0.015	0.008
Escherichia coli	NCTC13476	IMP-1	1	0.03	0.015	0.015
Klebsiella pneumoniae	ATCC BAA-1705	KPC-2	4	0.015	0.015	0.015
Klebsiella pneumoniae	ATCC BAA-1902	KPC-3	64	0.12	0.12	0.06
Klebsiella pneumoniae	ATCC BAA-1903	KPC-2	4	0.015	0.015	0.015
Klebsiella pneumoniae	ATCC BAA-1905	KPC-2	8	0.015	0.015	0.015
Klebsiella pneumoniae	ATCC BAA-2344	KPC-2	4	0.015	0.015	0.015
Klebsiella pneumoniae subsp. pneumoniae	ATCC BAA-2470	NDM-1	16	0.03	0.03	0.03
Klebsiella pneumoniae subsp. pneumoniae	ATCC BAA-2524	OXA-48	0.25	0.03	0.03	0.03
Klebsiella pneumoniae	NCTC13439	VIM-1	0.5	0.03	0.03	0.03
Klebsiella pneumoniae	NCTC13440	VIM-1	0.5	0.03	0.03	0.03
Klebsiella pneumoniae	KUB3163	IMP-1	0.5	0.03	0.015	0.015
Klebsiella pneumoniae	KUB3164	IMP-6	2	0.5	0.5	0.12
Enterobacter cloacae	KUB3165	IMP-34	0.25	0.03	0.03	0.015
Klebsiella pneumoniae	KUB3166	IMP-1	0.5	0.03	0.03	0.015
Enterobacter cloacae	KUB3167	IMP-1	0.25	0.03	0.03	0.015
Enterobacter cloacae	KUB3168	-	0.06	0.03	0.03	0.03
Serratia marcescens	KUB3169	Unidentified	2	1	0.25	0.12
Serratia marcescens	KUB3170	Unidentified	2	1	0.25	0.12
Serratia marcescens	KUB3171	-	2	2	2	2
Escherichia coli	KUB3605	NDM-1	32	0.015	0.015	0.015
Klebsiella pneumoniae	KUB3606	KPC-38	256	32	0.5	0.25
Klebsiella pneumoniae	KUB3607	IMP-1	0.5	0.03	0.03	0.015
Enterobacter cloacae	KUB3609	IMP-1	16	8	4	2
Enterobacter cloacae	KUB3610	IMP-1	4	4	4	4
Klebsiella aerogenes	KUB3614	-	2	0.5	0.5	0.25
Klebsiella aerogenes	KUB3615	-	0.12	0.03	0.015	0.015
Enterobacter cloacae	KUB3620	-	16	8	8	2
Klebsiella pneumoniae	KUB3628	IMP-1	8	0.25	0.12	0.06

Organism	Origin	Harbored carbapenemase genes	MIC of meropenem (μg/mL)			
			Meropenem	Meropenem/ KSP-1007 4 μg/mL	Meropenem/ KSP-1007 8 μg/mL	Meropenem/ KSP-1007 16 μg/mL
Klebsiella pneumoniae	KUB3629	IMP-1	0.5	0.03	0.015	0.015
Klebsiella pneumoniae	KUB3630	IMP-1	1	0.015	0.015	0.015
Klebsiella pneumoniae	KUB3631	IMP-1	4	0.12	0.06	0.03
Klebsiella pneumoniae	KUB3633	IMP-6	1	0.5	0.25	0.25
Klebsiella pneumoniae	KUB3634	IMP-6	1	0.5	0.25	0.12
Klebsiella pneumoniae	KUB3635	IMP-1	32	16	16	8
Klebsiella pneumoniae	ATCC BAA-1898	KPC-2	32	0.03	0.03	0.015
Klebsiella pneumoniae	ATCC BAA-1899	KPC-2	8	0.015	0.03	0.015
Klebsiella pneumoniae	ATCC BAA-1900	KPC-3	8	0.015	0.03	0.03
Klebsiella pneumoniae	ATCC BAA-1904	KPC-3	2	0.03	0.03	0.015
Klebsiella pneumoniae	ATCC BAA-2078	KPC-3	4	0.015	0.015	0.015
Enterobacter hormaechei	ATCC BAA-2082	KPC-2	16	0.12	0.06	0.03
Enterobacter cloacae	ATCC BAA-2341	KPC-3	16	0.03	0.03	0.03
Klebsiella pneumoniae	ATCC BAA-2342	KPC-3	4	0.015	0.015	0.015
Klebsiella pneumoniae	ATCC BAA-2343	KPC-3	16	0.03	0.03	0.03
Escherichia coli	ATCC BAA-2452	NDM-1	1	0.015	0.015	0.015
Enterobacter cloacae	ATCC BAA-2468	NDM-1	128	128	8	2
Escherichia coli	ATCC BAA-2471	NDM-6	32	0.12	0.12	0.06
Klebsiella pneumoniae subsp. Pneumoniae	ATCC BAA-2472	NDM-1	128	64	16	1
Klebsiella pneumoniae	ATCC BAA-2473	NDM-1	64	16	4	0.25
Providencia rettgeri deposited as P. rettgeri	ATCC BAA-2525	OXA-181	2	0.12	0.12	0.12
Klebsiella pneumoniae	ATCC BAA-2146	NDM-1	64	0.5	0.12	0.06

Table S3. The effects of 4% HSA on KSP-1007 or xeruborbactam with meropenem against CPE and CPAB

CPE (Carbapenemase)	MICs of meropenem (μg/mL)						CPAB (Carbapenemase)	MICs of meropenem (μg/mL)					
	Meropenem		Meropenem/ KSP-1007 4 μg/mL		Meropenem/ xeruborbactam 4 μg/mL			Meropenem		Meropenem/ KSP-1007 4 μg/mL		Meropenem/ xeruborbactam 4 μg/mL	
	0%*	4%†	0%*	4%†	0%*	4%†		0%*	4%†	0%*	4%†	0%*	4%†
E. coli CDC-137 (NDM-6)	64	128	0.25	0.25	0.25	≥8	CDC-33 (NDM-1, OXA-94)	128	128	8	8	16	32
E. coli CDC-150 (NDM-5)	128	64	0.03	0.06	0.12	4	CDC-37 (NDM-1, OXA-94)	128	128	4	4	8	32
E. coli CDC-162 (NDM-7)	128	128	0.12	0.12	0.12	≥8	CDC-83 (NDM-1, OXA-23, 69)	256	64	16	16	1	32
E. coli CDC-118 (NDM-1)	128	64	0.06	0.12	0.25	2	CDC-88 (NDM-1, OXA-64)	256	128	4	4	8	32
E. coli CDC-119 (NDM-1)	32	32	0.06	0.06	0.06	1	CDC-78 (OXA-71)	64	32	16	32	2	32
E. coli CDC-128 (NDM-1)	128	128	0.06	0.12	0.25	≥8	CDC-101 (OXA-24, 65)	128	128	8	16	2	32
E. coli NCTC 13476 (IMP-1)	4	4	0.03	0.06	0.015	0.12	CDC-291 (OXA-23, 66)	32	32	2	4	0.5	2
K. pneumoniae CDC-49 (NDM-1)	128	64	0.5	1	0.5	≥8	CDC-292 (OXA-66, 72)	256	256	128	128	1	128
K. pneumoniae ATCC BAA-2470 (NDM-1)	16	16	0.06	0.06	0.06	0.5	CDC-303 (OXA-23, 66)	32	16	2	1	0.25	1
K. pneumoniae NCTC13440 (VIM-1)	8	16	0.06	0.06	0.015	0.06	CDC-304 (OXA-66, 72)	64	64	2	8	0.5	2
E. cloacae CDC-154 (VIM-1)	4	8	0.06	0.12	0.03	0.06	CDC-309 (OXA-23, 82)	64	64	16	16	2	8
C. freundii CDC-157 (NDM-1)	64	64	0.25	0.5	0.25	4	CDC-313 (OXA-23, 69)	16	16	1	2	0.25	2
Geometric Mean	38.1	38.1	0.0857	0.128	0.0969	1.32	Geometric Mean	85.4	64.0	6.35	8.48	1.50	12.0

*: Standard condition, †: in the presence of 4% HSA

Table S4. The MICs of KSP-1007 and MEM against reference strains of aerobic bacteria

Reference strain/Note	MIC (μg/mL)	
	KSP-1007	Meropenem
Staphylococcus aureus ATCC 6538P/methicillin-sensitive	8	0.06
Staphylococcus aureus KUB4690/methicillin-resistant	>128	32
Staphylococcus epidermidis KUB4871/methicillin-sensitive	4	0.06
Staphylococcus epidermidis KUB4862/methicillin-resistant	>128	4
Staphylococcus lugdunensis KUB4804	4	0.12
Staphylococcus saprophyticus KUB4845	4	0.12
Micrococcus luteus ATCC 9341	128	0.06
Enterococcus faecalis ATCC 29212	64	1
Enterococcus faecium NCTC 12204	>128	32
Streptococcus pneumoniae ATCC 49619	2	0.06
Streptococcus pyogenes ATCC 12344	4	≤0.03
Streptococcus agalactiae ATCC 13813	4	≤0.03
Streptococcus mitis GTC495 (ATCC 49456)	4	≤0.03
Streptococcus mutans GTC218	8	≤0.03
Streptococcus porcinus ATCC 12391	2	≤0.03
Streptococcus pseudoporcinus ATCC BAA-1381	1	≤0.03
Streptococcus salivarius GTC215	0.25	≤0.03
Escherichia coli ATCC 25922	128	≤0.03
Klebsiella pneumoniae ATCC 700603	>128	≤0.03
Klebsiella aerogenes NCTC 10006 (ATCC 13048)	>128	≤0.03
Klebsiella oxytoca KUB4313	128	≤0.03
Acinetobacter calcoaceticus NBRC12552 (ATCC 14987)	>128	0.12
Acinetobacter baumannii ATCC 19606	>128	0.5
Stenotrophomonas maltophilia KUB3619	>128	>32
Pseudomonas aeruginosa ATCC 27853	>128	0.25
Citrobacter freundii ATCC 8090	128	≤0.03
Providencia rettgeri ATCC 9250	>128	0.06
Enterobacter hormaechei ATCC BAA-2082	>128	4
Enterobacter cloacae NBRC 13535 (ATCC 13047)	>128	0.12
Proteus mirabilis NBRC 3849 (NCTC 6197)	>128	≤0.03
Proteus vulgaris ATCC 6380	>128	≤0.03
Serratia marcescens NBRC 12648 (ATCC 14756)	>128	≤0.03
Moraxella catarrhalis ATCC 25238	64	≤0.03

Table S5. The MICs of KSP-1007 and metronidazole against reference strains of anaerobic bacteria

Reference strain	MIC (μg/mL)	
	KSP-1007	Metronidazole
Clostridioides difficile ATCC 700057	>256	0.5
Clostridioides difficile ATCC 43255	>256	1
Clostridioides difficile ATCC BAA-1803	>256	2
Clostridioides difficile ATCC BAA-1870	>256	1
Clostridium perfringens ATCC 13124	2	2
Peptostreptococcus anaerobius ATCC 27337	>256	0.5
Peptoniphilus asaccharolyticus ATCC 14963	32	1
Parvimonas micra KUB3000	64	0.5
Finegoldia magna ATCC 29328	128	2
Eggerthella lenta ATCC 43055	>256	1
Olsenella uli KUB3012	64	4
Bulleidia extructa KUB3013	32	2
Atopobium rimae KUB3014	8	1
Propionibacterium acnes ATCC 11827	32	>64
Actinomyces odontolyticus ATCC 17929	256	>64
Bacteroides fragilis ATCC 25285	8	1
Bacteroides fragilis KUB2982	16	2
Bacteroides fragilis KUB2983	16	0.5
Bacteroides thetaiotaomicron ATCC 29741	64	2
Bacteroides thetaiotaomicron KUB2984	32	1
Bacteroides ovatus KUB3009	128	1
Prevotella histicola KUB2989	8	2
Prevotella intermedia KUB2991	4	1
Prevotella melaninogenica KUB3005	4	1
Prevotella buccae KUB2992	8	2
Fusobacterium varium ATCC 27725	>256	0.5
Fusobacterium nucleatum ATCC 25586	32	≤0.125
